# Effect of propofol and sevoflurane anesthesia on the optic nerve sheath: systematic review and meta-analysis

**DOI:** 10.1016/j.bjane.2025.844646

**Published:** 2025-06-04

**Authors:** Rodolfo Otávio Tomaz Bertti, Luiz Antonio Vane, José Mariano Soares de Moraes, Paulo do Nascimento Junior, Lucas Fachini Vane, Norma Sueli Pinheiro Módolo, Matheus Fachini Vane

**Affiliations:** a Universidade Estadual Paulista (UNESP), Departamento de Especialidades Cirúrgicas e Anestesiologia, Botucatu, SP, Brazil; b Faculdade de Medicina de Juiz de Fora, Juiz de Fora, MG, Brazil; c Faculdade de Ciências Médicas de São José dos Campos, Bioestatística, Humanitas, SP, Brazil; d Universidade de São Paulo (USP), Hospital das Clínicas, Anestesiologista, São Paulo, SP, Brazil

**Keywords:** Intracranial pressure, Laparoscopy, Optic nerve, Propofol, Robotic surgical procedures, Sevoflurane

## Abstract

**Background:**

To facilitate the surgical view, laparoscopic and robotic pelvic surgeries require a pneumoperitoneum with the Trendelenburg position, which may result in elevated Intracranial Pressure (ICP). The choice of anesthetic agents may also influence ICP. Ultrasonographic evaluation of the Optic Nerve Sheath Diameter (ONSD) is a promising way to evaluate ICP. In this systematic review, we aimed to evaluate the ONSD, as an indirect estimation of ICP, in patients undergoing laparoscopic/robotic surgeries under pneumoperitoneum and Trendelenburg position.

**Methods:**

A literature search was performed to identify prospective randomized clinical trials in which the primary endpoint was the evaluation of the ONSD using sevoflurane or propofol anesthesia after the onset of pneumoperitoneum and Trendelenburg position. The mean and the standard deviation of the ONSD in each intervention group were extracted from the included trials for analysis. Mean difference with 95% Confidence Interval (95% CI) was calculated.

**Results:**

Five randomized controlled trials, with 277 subjects, were allocated to this study. Compared with the baseline, there was an increase in ONSD from 0.5h to 3 hours (p < 0.05) in both propofol and sevoflurane groups. Furthermore, propofol reduced the ONSD compared to sevoflurane (mean difference: -0.23 mm, 95% CI: -0.37 to -0.10; studies = 5; I^2^ = 23%).

**Conclusion:**

There is evidence indicating, through ultrasonographic analysis of the ONSD, that propofol probably reduces ICP compared to sevoflurane in robotic and laparoscopic pelvic surgeries.

## Introduction

Every three years, the prevalence of cancer in the United States is estimated using the Surveillance, Epidemiology, and End Results Cancer Registries (SEER) database. In 2022, more than 3.5 million North American men were affected by prostate cancer and 268490 new patients were diagnosed with the disease.^1^ Minimally invasive radical prostatectomies first occurred in the 1990s.^2^ The “da Vinci” robotic platform became the available model most used by doctors in the world today.^3^ In the USA, more recent data indicate that more than 85% of radical prostatectomies are performed with robotics (RARP).^4^

In the past 20-years, pelvic laparoscopic and robotic surgeries have become the method of choice due to their oncological benefits and the reduction in perioperative complications. They were previously performed using open techniques. However, these procedures require the establishment of pneumoperitoneum with Carbon Dioxide (CO_2_) and Trendelenburg position, which lead to physiological changes in the cardio-respiratory system and impact on Intracranial Pressure (ICP). Pulmonary and cardiovascular repercussions are known and monitored. On the other hand, the traditional measurement of ICP proved to be an impracticable magnitude to be implemented perioperatively due to its complexity. In the past two decades, studies that measure the diameter of the Optic Nerve Sheath (ONSD) by ultrasonography have proven to be an easy-to-implement and noninvasive approach to detect ICP.^5^, ^6^, ^7^

Anesthetic agents may influence ICP during surgery. Under propofol anesthesia, a decrease in cerebral blood flow, cerebral metabolic rate, and ICP has been reported.^8^, ^9^, ^10^ Conversely, sevoflurane is a vasodilator with the potential to increase ICP.^11^ The effects of anesthetics on ICP, through pneumoperitoneum and the steep Trendelenburg position, require more in-depth knowledge. The objective of this systematic review is to investigate whether there is a difference between the anesthetics propofol and sevoflurane regarding the diameter of the optic nerve sheath, measured by ocular ultrasonography, as an indirect predictor of ICP, in simple laparoscopic and robotic pelvic surgeries.

## Methods

The study was registered in PROSPERO, CRD42023387503 (www.crd.york.ac.uk/Prospero), with the planned analyses performed. The PICO Diagram^12^ listed is P: Adult patients undergoing surgery in the Trendelenburg position and use; I: Anesthetic maintenance with propofol; C: Anesthetic maintenance with sevoflurane; O: Ultrasound diameter of the optic nerve sheath; S: Randomized Controlled Clinical Trials (RCTs).

Criteria for inclusion: patients between 19 and 79 years of age with an American Society of Anesthesiologists (ASA) physical status of I–III who were underwent elective laparoscopic or robot-assisted pelvic surgery. Patients with a previous neurological disease or cerebrovascular disease that could increase ICP, history of allergy to anesthetic drugs, pregnancy and patients with a history of ophthalmological disease were excluded.

A timeless search strategy with high sensitivity and moderate specificity and precision was developed between November 2021 and December 2022, according to the Cochrane Manual for Systematic Reviews of Interventions, version 6.3.^13^ STRING was built through an advanced search on the leading web platforms: PubMed; Embase; Cochrane; Virtual Health Library (VHS) Portal. Other sources, sites, and meta-search tools were part of the strategy: Handsearch (manual search); Grey Literature (Wordwidescience.org, Qinsight, Oasis.br, Grey Literature Report); Preprints: MedRxiv, Scielo preprint; Tripdatabase, ClinicalTrail.gov (ongoing clinical trials, records); University of York; Scielo; BMJ Clinical Evidence; Epistemonikos; Scopus; CINAHL (Cumulative index to nursing and allied health literature); BDTD (Portal of the Digital Library of Theses and Dissertations at USP); and Google Scholar (Appendix 1).

The searched terms and descriptors were: “surgery”, “Trendelenburg position or cephalo-declined”, and “intracranial pressure”. The MeSH terms used were: “Head-Down Tilt”, “Trendelenburg Position”, “Surgery”, “Surgery, General”, “Surgical Procedures, Operative”, “Intracranial Pressure”, “Intracranial Hypertension”, and “Papilledema”.

**Figure 1:**
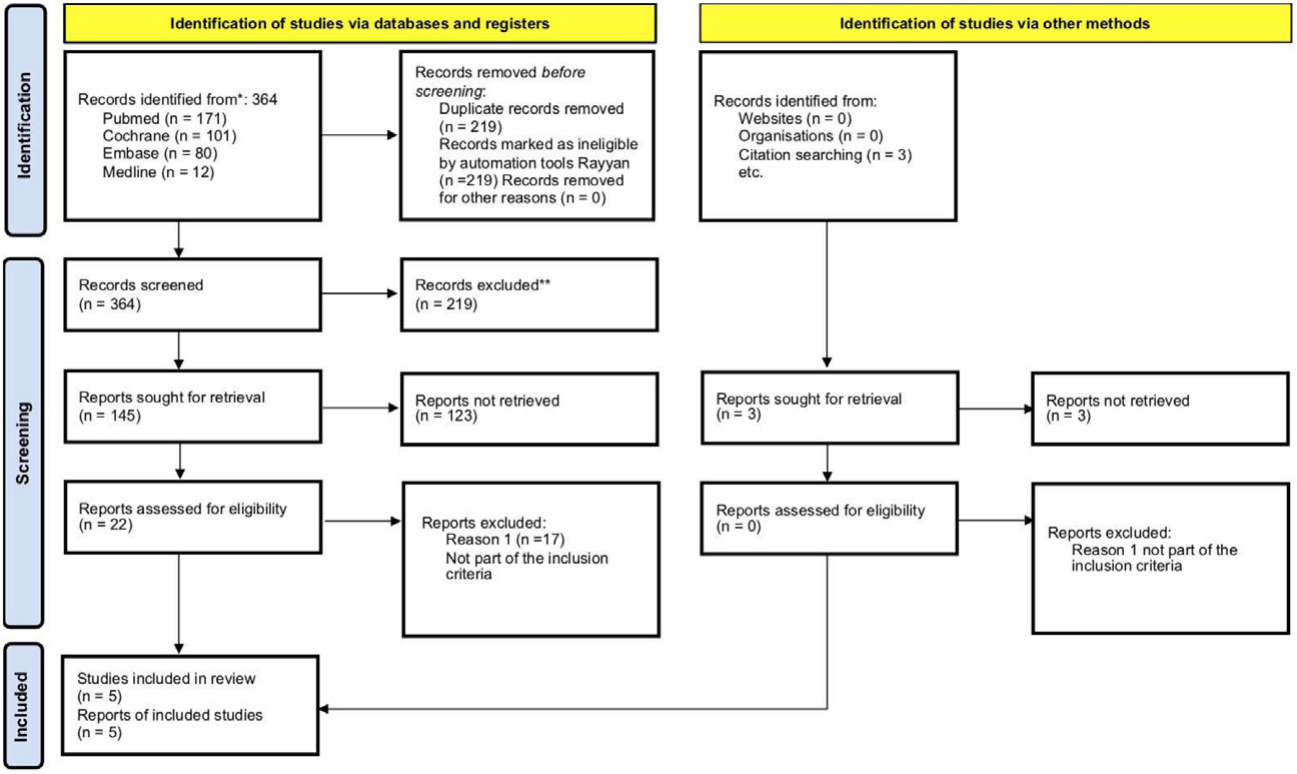
PRISMA 2020, Flow diagram for new systematic reviews, which included searches of databases, registers and other sources.

The studies were filtered to include all randomized clinical articles in English, Portuguese, Spanish, and others (Fig. 1). Two independent authors (VTC and NCJ) analyzed all relevant studies for selection and data extraction, and the discrepancies were solved by a third author (MFV). Data was managed using the Rayyan *software*.^10^^,^^14^, ^15^, ^16^, ^17^

Data were extracted from methods (study design and definition), identification data (source of sponsorship, country, and details of authors such as name, institution, e-mail, and address), data on the characteristics of the participants (number of randomized participants, number of assessed participants, and number lost from follow-up with reasons described, basic data characteristics, inclusion criteria), interventions (number of participants within each intervention group and intervention description), outcome measures (type of outcome report, breadth, measurement unit, direction, and observations), study design characteristics (and risk of bias assessment), and any other relevant information.

Two authors (VTC and NCJ) independently assessed the risk of bias of each included study using version 2 of the Cochrane’ Risk of Bias’ tool (RoB2) according to the recommendations in the Cochrane Handbook for Systematic Reviews of Interventions version 6.3.^13^

**Figure 2:**
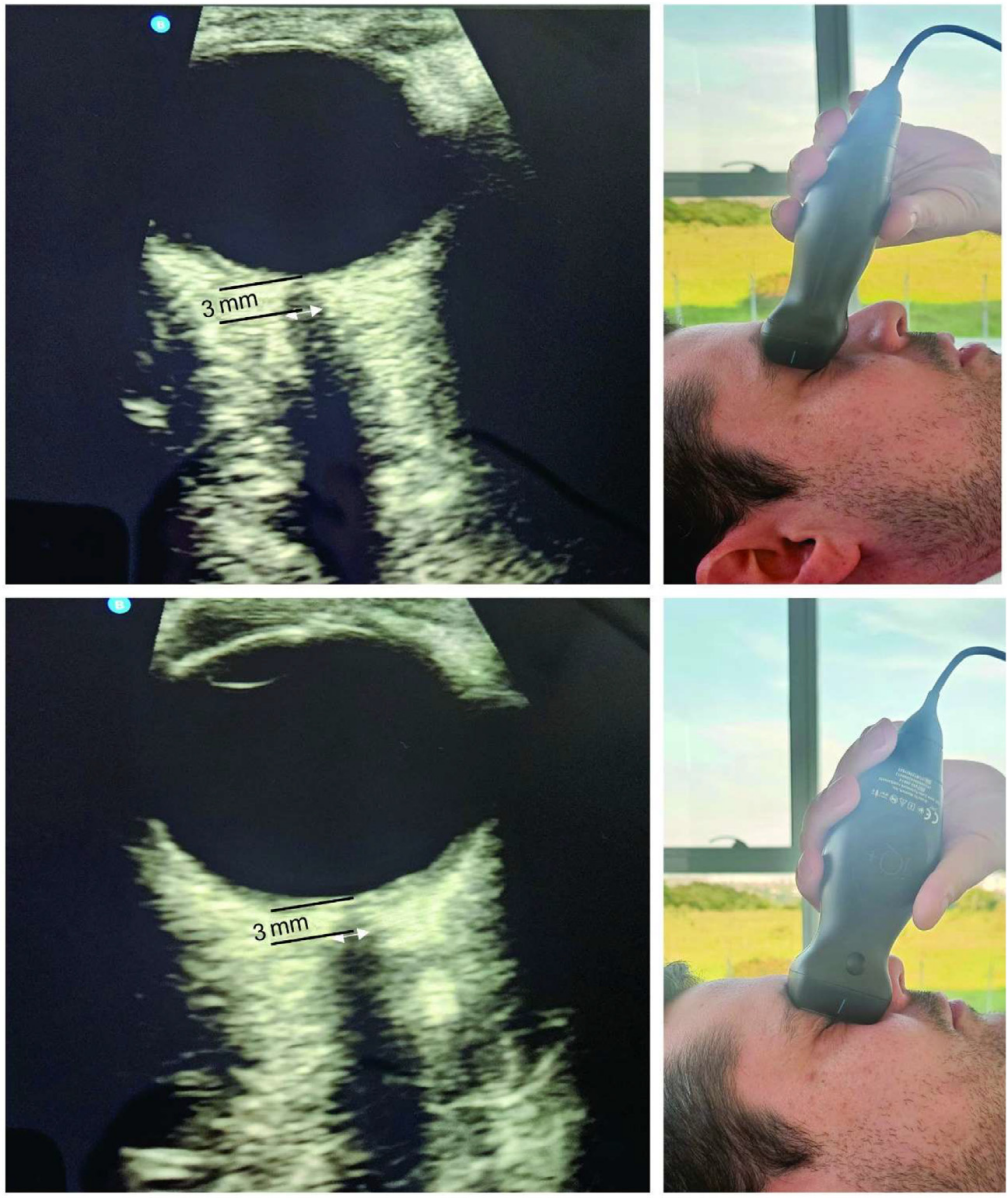
Ocular ultrasound: 3 views [transverse (top), sagittal (middle) and bottom (bottom)], diameter of red optic nerve sheath (inside diameter), diameter of yellow optic nerve sheath (outside diameter), green distance 3 mm behind the globe (Kishk and Ebraheim, 2019; Raval et al., 2020).

The primary outcome was the change in ONSD, measured by ultrasound^18^ from 0.5h to 3 hours after the onset of pneumoperitoneum and cephalon decline compared to the baseline values after anesthesia (Fig. 2). The mean, Standard Deviation (SD), and number of participants in each intervention group from the included trials were extracted for continuous data. When trials reported any other measure of dispersion (e.g., confidence interval or standard error), the SD was calculated according to the instructions in the Cochrane Handbook for Systematic Reviews of Interventions version 6.3.13 Data extraction was based on intention-to-treat analysis when possible. The data were summarized using meta-analysis, the inverse generic variance method and the random effects model since one of the studies presented the results separated by eye, which were later grouped following the instructions in the Cochrane Handbook for Systematic Reviews of Interventions version 6.313. In the present study, a cut-off value of 5 mm was used to predict a high ICP (above 20 mmHg).

The analysis unit considered was each individual (randomization was used from each participant, individually). When several points in time were reported in the same study, the data were related to the most extended follow-up of the surgical time the patient was undergoing pneumoperitoneum (the exception was the study by Sujata et al*.*,^14^ in which the extracted data concerned the highest value found, due to data availability).

Possible clinical heterogeneity was assessed considering participants, interventions, outcomes, and study characteristics for the included trials. Statistical heterogeneity was visually inspected in the forest plots, and the Chi^2^ test was used. In addition, the I^2^ statistic was used to describe the proportion of variation in the effect estimates that is due to variability between studies and not to sampling error, following the recommendations of chapter 10 of the Cochrane Handbook for Systematic Reviews of Interventions version 6.3.^13^

Regarding the assessment of the certainty of the evidence, two authors (VTC and NCJ) used the approach proposed by the GRADE Working Group and the recommendations of chapter 14 of the Cochrane Handbook for Systematic Reviews of Interventions version 6.3.^13^ The GradePro GDT software was used to analyze the overall certainty of the evidence. The investigation of publication bias was planned with the funnel chart. The subgroup and sensitivity analysis was designed for studies with high risk of bias.

## Results

A total of 277 patients were allocated to this study. The sample consisted of 16 women undergoing robotic gynecological surgery and 115 men, in whom robotic-assisted radical prostate surgery predominated (Table 1). Weight and age were presented as mean ± standard deviation. There were no statistically significant differences between the propofol and sevoflurane groups (p > 0.05) regarding the variables number of patients, weight and age (Table 2). In studies E2 and E3, lower mean ages were observed in the propofol and sevoflurane groups compared to the other articles.

**Table 1:** Identification of studies.

Study	Source/Type	Title	Surgery	Authors	DOI https://doi.org/
E1	J Robot Surg (2019) RCT	A randomized trial to compare the increase in intracranial pressure as correlated with the optic nerve sheath diameter during propofol versus sevoflurane-maintained anesthesia in robotic-assisted laparoscopic pelvic surgery	RAGS, RAGS	Sujata N. et al.^14^	10.1007/s11701-018-0849-7
E2	BMC Anesthesiol (2021) RCT	Effects of sevoflurane and propofol on the optic nerve sheath diameter in patients undergoing laparoscopic gynecological surgery: a randomized controlled clinical study	LGS	Geng W. et al.^15^	10.1186/s12871-021-01243-7
E3	Anesth Pain Med (2019) RCT	Optic nerve sheath diameter changes during gynecologic surgery in the Trendelenburg position: comparison of propofol- based total intravenous anesthesia and sevoflurane anesthesia	RAGS	Lee YY. et al.^16^	10.17085/apm.2019.14.4.393
E4	Biomed Res Int (2019) RCT	Kim Y, Choi S, Kang S, Park B. Propofol Affects Optic Nerve Sheath Diameter Less than Sevoflurane during Robotic Surgery in the Steep Trendelenburg Position	RARP	Kim Y. et al.^10^	10.1155/2019/5617815
E5	BMC Anesthesiol (2018) RCT	Propofol attenuates the increase of sonographic optic nerve sheath diameter during robot- assisted laparoscopic prostatectomy: a randomized clinical trial	RARP	Yu J. et. al.^17^	10.1186/s12871-018-0523-7

DOI, Digital Object Identifier; RAGS, Robotic-Assisted Gynecologic Surgery; RARC, Robotic-Assisted Radical Cystectomy; LGS, Laparoscopic Gynecologic Surgery; RARP, Robotic-Assisted Radical Prostatectomy; RCT, Randomized Clinical Trail.

**Table 2:** Demographic data.

	N total	N PROP	N SEVO	Age (years) PROP Mean ± SD	Age (years) SEVO Mean ± SD	Weight (kg) PROP Average ± SD	Weight (kg) SEVO Average ± SD
**E1**	49	25 (1 ♀)	24 (1 ♀)	62.88 ± 8.14	65.33 ± 8.51	72.56 ± 9.78	78.54 ± 14.84
**E2**	110	55 ♀	55 ♀	40.53 ± 1.08	41.15 ± 10.26	59 (54.5, 63)	56 (51.9, 60)
**E3**	50	25 ♀	25♀	45 ± 13.80	44 ± 11.90	58 ± 6.80	56 ± 9.80
**E4**	32	16 ♂	16 ♂	64.38 ± 7.86	68.44 ± 7.97	69.38 ± 10.25	66.69 ± 8.65
**E5**	36	18 ♂	18 ♂	66.1 ± 7.20	63.6 ± 7.90	72.3 ± 6.50	69.80 ± 10.60

PROP, Propofol; SEVO, Sevoflurane; SD, Standard deviation; ♀, Female; ♂, Male.

Anesthetic and surgical data were recorded during the perioperative period following the parameters: fluid volume; physical status according to the American Society of Anesthesiology (ASA); Peak Inspiratory Pressure (PIP); Blood Pressure (BP); end-tidal carbon dioxide (ETCO_2_); end-tidal effect-site concentration (Ce); incline (all parameters p > 0.05). The only exception occurred with the heart rate variable, which showed higher levels in the sevoflurane group in studies E3 and E5.

The ONSD of the groups are shown in Table 3. Although E1 recruited patients with console time < 5 hours, the period analyzed to search for effects on ONSD reached comparisons up to 180 minutes after anesthetic induction due to the consistency of the information provided by the included articles.

**Table 3:** Comparative data of optic nerve sheath diameters.

Studies	POST-IND SDPROP (mm)	POST-IND SDSEVO (mm)	Ta10 min SDPROP (mm)	Ta10 min SDSEVO (mm)	Ta30 min SDPROP (mm)	Ta30 min SDSEVO (mm)	Ta60 min SDPROP (mm)	Ta60 min SDSEVO (mm)	Ta120 min SDPROP (mm)	Ta120 min SDSEVO (mm)	Ta180 min SDPROP (mm)	Ta180 min SDSEVO (mm)
E1	3.6 ± 0.3	3.5 ± 0.3	‒	‒	3.8	3.8	3.8	3.8	3.8	3.9	3.8 ± 0,4	4.1 ± 0.4
E2	4.06 ± 0.45	4.05 ± 0.45	4.64 ± 0.48	4.50 ± 0.29	4.77 ± 0.45	4.62 ± 0.28	4.83 ± 0.43	4.66 ± 0.28	4.82 ± 0.41	4.71 ± 0.28	‒	‒
E3	4.6	4.7	4.9	5.1	5.15	5.20	‒	‒	‒	‒	‒	‒
E4	3.6 ± 0.32	3.6 ± 0.16	‒	‒	3.73 ± 0.23	4.0 ± 0.23	3.6 ± 0.29	3.8 ± 0.2	‒	‒	3.62 ± 0.52	3.89 ± 0.3
E5	4.75 ± 0.37	4.74 ± 0.42	‒	‒	5.22 ± 0.34	5.42 ± 0.36	5.27 ± 0.35	5.57 ± 0.28	‒	‒	‒	‒

POST-IND, Post-anesthetic Induction; SDPROP, Sheath Diameter with Propofol; SDSEVO, Sheath Diameter with Sevoflurane; Ta, Anesthesia time.

When comparing the baseline optic nerve sheath diameters at anesthetic induction in relation to the values achieved after the introduction of pneumoperitoneum and head tilt, in all articles statistical significance was reached regarding the effect of increasing ONSD, regardless of the type of anesthetic used, considering the mean values ± SD. Also in the studies, results in ONSD were found with a statistically significant difference between the groups after establishing pneumoperitoneum with carbon dioxide and the Trendelenburg position (p < 0.05), except for study E3.

ONSD variation over time, which exceeded the limit value of 5 mm (presumed ICP > 20 mmHg), was observed in the sevoflurane group in E1, E3, and E5.

**Figure 3:**
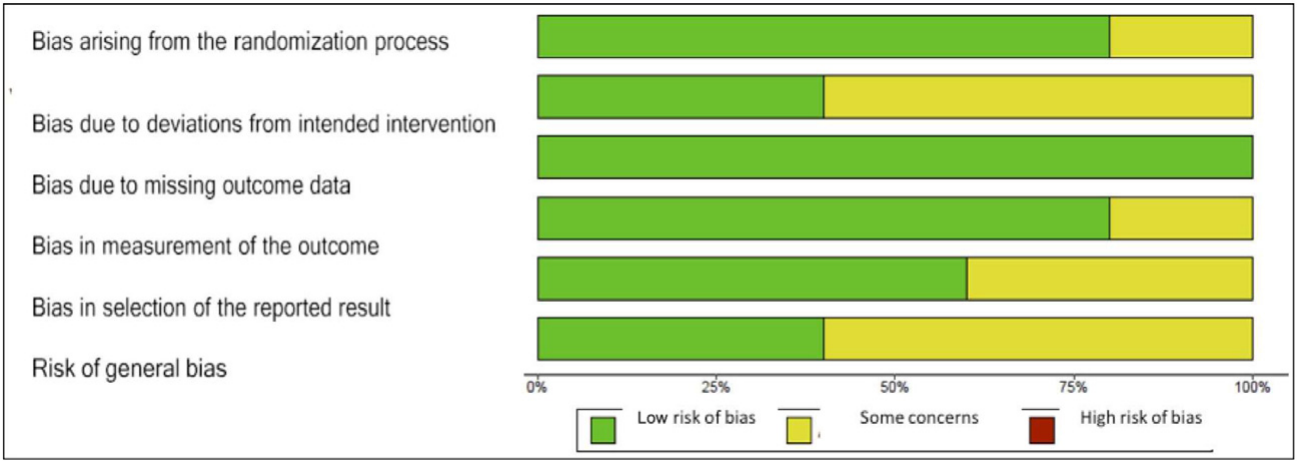
Risk of general bias of studies included in the systematic review.

The risk of bias assessments for each included study was directly expressed in the meta-analysis, and the decisions considering the different types of bias are presented in Figure 3.

Applying the GRADE criteria, we found moderately certain evidence that propofol probably slightly reduces intracranial pressure compared to sevoflurane (lowered once due to imprecision). Publication bias assessment was not possible since less than ten trials were included in the meta-analysis. Sensitivity and subgroup analyses were not required considering no study was assessed as a high risk of general bias.

**Figure 4:**
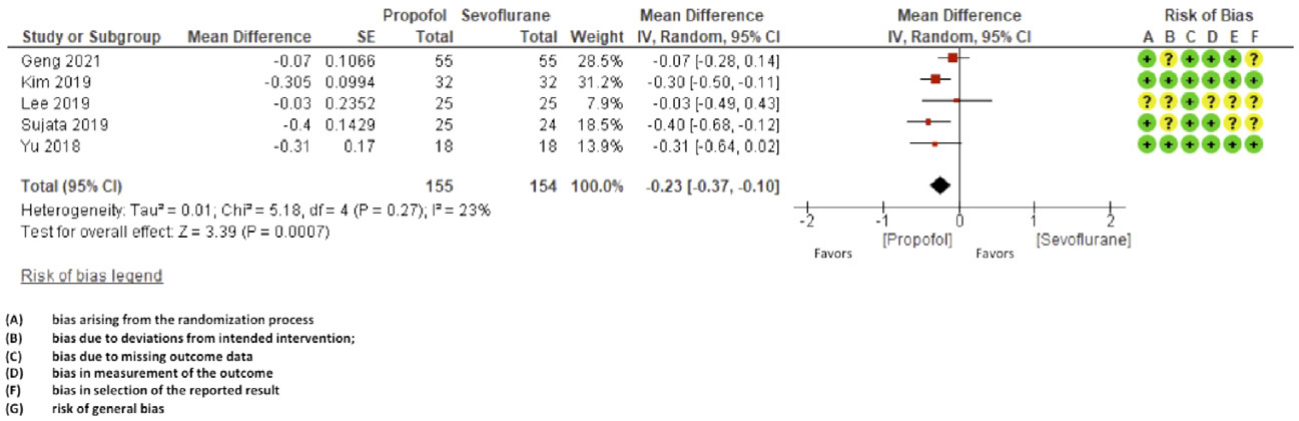
Forest plot of the comparison: propofol versus sevoflurane, outcome: intracranial pressure and GRADE.

Although E1 recruited patients with < 5 hours console time, the analyzed period used to search for effects on ONSD reached comparisons up to 180 minutes after anesthetic induction. When comparing the basal optic nerve sheath diameters in anesthetic induction concerning the values achieved after the introduction of pneumoperitoneum and head-declined position, all articles showed statistical significance regarding the effect of increasing ONSD, regardless of anesthetic type, considering the mean values ± SD. Propofol causes less elevation intracranial pressure compared to sevoflurane (mean difference: -0.23 mm, 95% CI -0.37 to -0.10; studies = 5; I^2^ = 23%) (Fig. 4).

A sensitivity and subgroup analysis was planned for studies with high risk of bias, however it was not necessary because no study was classified as having high risk of bias overall. Furthermore, due to the low importance of the heterogeneity found in the articles, the investigation was once again shown to be unnecessary.

## Discussion

Robot-assisted prostatectomy has become the dominant surgical approach for prostate cancer treatment, mainly in developed countries. More recent data indicate that more than 85% of radical prostatectomies in the United States are performed laparoscopically or are robotically-assisted.^4^ RARP - robotic-assisted radical prostatectomy – allows for many benefits when we consider oncological outcomes and perioperative complications compared to open surgery. Thus, new challenges arise concerning understanding the impact on the physiological phenomena related to the method. Despite the technique’s effectiveness, research is necessary to ensure greater safety for surgical patients.

ONSD ultrasonographic measurement has been known as a simple and non-invasive surrogate instrument for ICP monitoring. A distensible subarachnoid space surrounds the retrobulbar optic nerve; therefore, the nerve sheath expands when the ICP rises.^19^ Mehrpaur et al.^20^ demonstrated that optic nerve sonography as a ONSD parameter is a real-time technique for detecting intracranial hypertension. A pooled sensitivity of 0.90 and pooled specificity of 0.85 were observed in the NO sonographic evaluation.^10^^,^^21^

As a technique, ocular ultrasonography can be quickly learned. Tayal et al.^22^ and Sujata et al.^14^ showed that, for an experienced operator, ten scans, including three abnormal scans, should be sufficient training to learn the method. At the same time, 25 ultrasounds may be necessary for new sonographers. In addition to these findings, both eyes are measured in less than 4 minutes. Just as anesthesiologists monitor a patient’s cardiorespiratory condition, they can learn to handle the ultrasound device in the operating room.

Trends in the diameter of the optic nerve sheath were investigated at different time intervals (0.5h to 3 hours) in the five publications considered for this systematic review. No statistically significant differences were identified between the groups (p > 0.05) in the various variables that affect the ICP. The only exception occurred with the heart rate variable, which showed higher levels in the sevoflurane group in studies E3 and E5, with no direct correlation with the increase in MAP and its fractions.

Pneumoperitoneum causes hypercapnia, cerebrovascular dilatation, increased intracranial blood flow, and increased intracranial pressure.^23^ While these effects rarely result in serious complications such as cerebral hemorrhage and edema, mild neurological complications, such as nausea, vomiting, and headaches, sometimes occur.^15^^,^^24^ Pandey et al.^24^ reported two cases of robotic radical cystectomy with perioperative neurological complications and documented neurological deterioration after extubation, probably due to cerebral edema. They suggested that the duration and placement be optimized. Weber et al.^25^ reported postoperative visual loss due to a prolonged steep Trendelenburg position during prostatectomy. Lee et al.^16^ considered that reversible neurological deficits, such as a transient ischemic attack, may not be detected due to a lack of postoperative course follow-up.

In studies E1, E3, E5, the 5 mm ONSD values measurements did not result in any adverse postoperative neurological or ophthalmological sequel. However, the absence of a specific population (with glaucoma, retinopathy, previous cerebrovascular diseases, with console time greater than 4 hours and age greater than 79 years) compromises the external validity for this population.

When applying the GRADE criteria for assessing the risk of bias in the studies, the result was categorized as a “moderate certainty of evidence”. The domains named as routes due to deviations from planned interventions and bias in results selection raised some concerns. Heterogeneity was present in the data used for the meta-analysis, but the value of I^2^ = 23% was regarded as unimportant (0 to 40%). Factors such as differences in patient characteristics, types of surgery, positions and angles, insufflation pressures, and the complex mechanisms for raising ICP and compensation may be considered possible causes for heterogeneity. In addition, the level of experience or skill in performing ocular sonography may affect measurement results in the ONSD. Possibly, this may explain the findings pointed out by Yu et al.^17^ (2018) and Lee et al.^16^ concerning larger measurements of ONSD at baseline and after anesthetic induction, reproducing high ICPs (ICP 20 mmHg and ONSD 5 mm).

Sevoflurane presents a dose-dependent effect on intrinsic cerebral vasodilation activity. The cerebral blood flow increases significantly, and, as a result, ICP may rise. On the other hand, propofol decreases cerebral metabolic rate and local blood perfusion, which causes less impact on ICP.^17^ Thus, ICP increases with both anesthetics. However, with a lower impact on ONSD, propofol reached a negative mean difference of -0.23 mm (0.37 less to 0.1 less) compared to sevoflurane.

Although there were differences in anesthetic techniques, Trendelenburg angles and surgery time, heterogeneity was low.

In short, anesthetic agents can influence ICP. The results obtained by this review favor the use of anesthetic maintenance with propofol. Through the concept of indirect evidence, it is also possible to expand the recommendation for the benefit of propofol to elderly patients (> 80-years-old) and/or with higher neurological and ophthalmological risks.

## Conclusion

There is evidence to demonstrate, through optic nerve sheath diameter ultrasonographic analysis, that propofol causes less elevation to intracranial pressure compared to sevoflurane in the context of pure robotic and laparoscopic pelvic surgery.

There are limitations regarding the number of primary studies, which makes it impossible to broaden the discussion about the safety of minimally invasive pelvic surgeries in more specific settings, such as elderly patients, patients with ophthalmopathy, and neuropathies. Further studies must be encouraged to overcome these uncertainties.

## Registry

PROSPERO: https://www.crd.york.ac.uk/PROSPERO/

#CRD42023387503

https://www.crd.york.ac.uk/PROSPERO/display_record.php?RecordID=387503

Date of first submission: 21 February 21^st^, 2023

Date of registry: 4 March 4^th^, 2023

## Authors’ contributions

Rodolfo Otávio Tomaz Bertti: Conception and design of the study; Acquisition of data, analysis and interpretation of data; Drafting and revising the manuscript.

Luiz Antonio Vane: Conception and design of the study, Acquisition of data, analysis and interpretation of data; Drafting and revising the manuscript; General supervision of the study.

José Mariano Soares de Moraes: Revising the manuscript.

Paulo do Nascimento Junior: Analysis and interpretation of data; Drafting and revising the manuscript.

Lucas Fachini Vane: Analysis and interpretation of data; Drafting and revising the manuscript.

Norma Sueli Pinheiro Módolo: Revising the manuscript.

Matheus Fachini Vane: Analysis and interpretation of data; drafting and revising the manuscript.

## Conflicts of interest

The authors declare no conflicts of interest.

## Appendix Supplementary materials

Image, application 1

## References

[1] Miller K.D., Nogueira L., Devasia T. (2022). Cancer treatment and survivorship statistics, 2022. CA Cancer J Clin.

[2] Schuessler W.W., Schulam P.G., Clayman R.V., Kavoussi LR. (1997). Laparoscopic radical prostatectomy: initial short-term experience. Urology.

[3] Liberman D., Trinh Q.D., Jeldres C., Zorn KC. (2012). Is robotic surgery cost-effective: yes. Curr Opin Urol.

[4] Faiena I., Dombrovskiy V.Y., Modi P.K. (2015). Regional Cost Variations of Robot-Assisted Radical Prostatectomy Compared With Open Radical Prostatectomy. Clin Genitourin Cancer.

[5] Launey Y., Nesseler N., Le Maguet P., Mallédant Y., Seguin P. (2014). Effect of osmotherapy on optic nerve sheath diameter in patients with increased intracranial pressure. J Neurotrauma.

[6] Major R., Girling S., Boyle A. (2011). Ultrasound measurement of optic nerve sheath diameter in patients with a clinical suspicion of raised intracranial pressure. Emerg Med J.

[7] Wang J., Li K., Li H. (2019). Ultrasonographic optic nerve sheath diameter correlation with ICP and accuracy as a noninvasive surrogate ICP measurement tool in patients with decompressive craniotomy. J Neurosurg.

[8] Banevičius G., Rugytė D., Macas A., Tamašauskas A., Stankevičius E. (2010). The effects of sevoflurane and propofol on cerebral hemodynamics during intracranial tumours surgery under monitoring the depth of anaesthesia. Medicina (Kaunas).

[9] Bundgaard H., von Oettingen G., Larsen K.M. (1998). Effects of sevoflurane on intracranial pressure, cerebral blood flow and cerebral metabolism. A dose-response study in patients subjected to craniotomy for cerebral tumours. Acta Anaesthesiol Scand.

[10] Kim Y., Choi S., Kang S., Park B. (2019). Propofol Affects Optic Nerve Sheath Diameter Less than Sevoflurane during Robotic Surgery in the Steep Trendelenburg Position. Biomed Res Int.

[11] Matta B.F., Heath K.J., Tipping K., Summers AC. (1999). Direct cerebral vasodilatory effects of sevoflurane and isoflurane. Anesthesiology.

[12] Akobeng AK. (2005). Principles of evidence-based medicine. Arch Dis Child.

[13] Higgins JPT, Thomas J, Chandler J, Cumpston M, Li T, Page MJ, Welch VA (editors). Cochrane Handbook for Systematic Reviews of Interventions. 2nd Edition. Chichester (UK): John Wiley & Sons, 2019.

[14] Sujata N., Tobin R., Tamhankar A., Gautam G., Yatoo AH. (2019). A randomised trial to compare the increase in intracranial pressure as correlated with the optic nerve sheath diameter during propofol versus sevoflurane-maintained anesthesia in robot-assisted laparoscopic pelvic surgery. J Robot Surg.

[15] Geng W., Chen C., Sun X., Huang S. (2021). Effects of sevoflurane and propofol on the optic nerve sheath diameter in patients undergoing laparoscopic gynecological surgery: a randomized controlled clinical study. BMC Anesthesiol.

[16] Lee Y.Y., Lee H., Park H.S., Kim W.J., Baik H.J., Kim DY. (2019). Optic nerve sheath diameter changes during gynecologic surgery in the Trendelenburg position: comparison of propofol-based total intravenous anesthesia and sevoflurane anesthesia. Anesth Pain Med.

[17] Yu J., Hong J.H., Park J.Y., Hwang J.H., Cho S.S., Kim YK. (2018). Propofol attenuates the increase of sonographic optic nerve sheath diameter during robot-assisted laparoscopic prostatectomy: a randomised clinical trial. BMC Anesthesiol.

[18] Raval R., Shen J., Lau D. (2020). Comparison of Three Point-of-Care Ultrasound Views and MRI Measurements for Optic Nerve Sheath Diameter: A Prospective Validity Study. Neurocrit Care.

[19] Kim E.J., Koo B.N., Choi S.H., Park K., Kim MS. (2018). Ultrasonographic optic nerve sheath diameter for predicting elevated intracranial pressure during laparoscopic surgery: a systematic review and meta-analysis. Surg Endosc.

[20] Mehrpour M., Oliaee Torshizi F., Esmaeeli S., Taghipour S., Abdollahi S. (2015). Optic nerve sonography in the diagnostic evaluation of pseudopapilledema and raised intracranial pressure: a cross-sectional study. Neurol Res Int.

[21] Dubourg J., Javouhey E., Geeraerts T., Messerer M., Kassai B. (2011). Ultrasonography of optic nerve sheath diameter for detection of raised intracranial pressure: a systematic review and meta-analysis. Intensive Care Med.

[22] Tayal V.S., Neulander M., Norton H.J., Foster T., Saunders T., Blaivas M. (2007). Emergency department sonographic measurement of optic nerve sheath diameter to detect findings of increased intracranial pressure in adult head injury patients. Ann Emerg Med.

[23] Blecha S., Harth M., Schlachetzki F. (2017). Changes in intraocular pressure and optic nerve sheath diameter in patients undergoing robotic-assisted laparoscopic prostatectomy in steep 45° Trendelenburg position. BMC Anesthesiol.

[24] Pandey R., Garg R., Darlong V., Punj J., Chandralekha, Kumar A. (2010). Unpredicted neurological complications after robotic laparoscopic radical cystectomy and ileal conduit formation in steep Trendelenburg position: two case reports. Acta Anaesthesiol Belg.

[25] Weber E.D., Colyer M.H., Lesser R.L., Subramanian PS. (2007). Posterior ischemic optic neuropathy after minimally invasive prostatectomy. J Neuroophthalmol.

