# Supplementary material for: Effect of propofol and sevoflurane anesthesia on the optic nerve sheath: systematic review and meta-analysis
Source: Braz J Anesthesiol. 2025 Jun 4;75(5):844646. doi: 10.1016/j.bjane.2025.844646 (PMC12268041; doi:10.1016/j.bjane.2025.844646)
Supplement: Supplementary file 1 [file mmc1.docx]

BJAN-D-24-0477_Supplementary Material

**Appendix 1**

**A - SEARCH STRATEGY**

- Cochrane Library;
- Medline (PubMed);
- Embase (Elsevier) – CAPES;
- Lilacs (BVS); - Handsearch;
- Grey Literature: WordWideScience.org, Qinsight, Oasis.br, Grey Literature Report;
- Preprints: MedRXIV, Scielo preprint; Tripdatabase, ClinicalTrail.gov;
- University of York;
- Scielo; - BMJ Clinical Evidence;
- Epistemonikos;
- Scopus;
- CINAHL (Cumulative index to nursing and allied health literature);
- BDTD (Portal of the Digital Library of Theses and Dissertations at USP).

**B -** **DESCRIPTORS– DeCS**

1. **Cirurgia**

| Descritor em português: | **Cirurgia Geral** |
| --- | --- |
| Descritor em inglês: | **General Surgery** |
| Descritor em espanhol: | **Cirugía General** |
| Descritor em francês: | **Chirurgie générale** |
| Termo(s) alternativo(s): | Cirurgia |
| Código(s) hierárquico(s): | H02.403.810.300 |
| Identificador Único RDF: | [https://id.nlm.nih.gov/mesh/](https://id.nlm.nih.gov/mesh/D013502) [D013502](https://id.nlm.nih.gov/mesh/D013502) |

| Descritor em português: | **Procedimentos Cirúrgicos Operatórios** |
| --- | --- |
| Descritor em inglês: | **Surgical Procedures, Operative** |
| Descritor em espanhol: | **Procedimientos Quirúrgicos Operativos** |
| Descritor em francês: | **Procédures de chirurgie opératoire** |
| Termo(s) alternativo(s): | Intervenção Cirúrgica Intervenções Cirúrgicas Operação Cirúrgica Operações Cirúrgicas Procedimento Cirúrgico  Procedimento Cirúrgico Operatório Procedimento Operatório Procedimentos Cirúrgicos  Procedimentos Operatórios |
| Código(s) hierárquico(s): | E04 VS3.003.001.006.002 |

1. **Trendelenburg ou cefalodeclive**

| Descritor em português: | **Decúbito Inclinado com Rebaixamento da Cabeça Termo(s) alternativo(s):**  Decúbito Inclinado com Cabeça para Baixo Posição de Trendelenburg |
| --- | --- |
| Descritor em inglês: | **Head-Down Tilt Termo(s) alternativo(s):** Head Down Tilt Position, Trendelenburg Tilt, Head-Down  Trendelenburg Position |

| Descritor em espanhol: | **Inclinación de Cabeza Termo(s) alternativo(s):**  Posición de Trendelenburg |
| --- | --- |
| Descritor em francês: | **Position déclive Termo(s) alternativo(s):** Décubitus déclive  Position de Trendelenburg Posture declive |

1. **Pneumoperitôneo**

Pneumoperitônio / Pneumoperitoneum / Neumoperitoneo

1. **Pressão Intracraniana**

| Descritor em português: | **Pressão Intracraniana** |
| --- | --- |
| Descritor em inglês: | **Intracranial Pressure** |
| Descritor em espanhol: | **Presión Intracraneal** |
| Descritor em francês: | **Pression intracrânienne** |
| Termo(s) alternativo(s): | Pressão Intracerebral Pressão Subaracnóidea |
| Código(s) hierárquico(s): | G11.561.170.505 |

1. **Nervo óptico ou disco óptico**

| Descritor em português: | **Nervo Oftálmico** |
| --- | --- |
| Descritor em inglês: | **Ophthalmic Nerve** |
| Descritor em espanhol: | **Nervio Oftálmico** |
| Descritor em francês: | **Nerf ophtalmique** |

| Descritor em português: | **Disco Óptico Termo(s) alternativo(s):** Cabeça do Nervo Óptico Mancha Cega  Papila Óptica |
| --- | --- |
| Descritor em inglês: | **Optic Disk** |
| Descritor em espanhol: | **Disco Óptico** |
| Descritor em francês: | **Papille optique** |

**C - HIGH SENSITIVITY SEARCH STRATEGIES**

Building the search STRING with high sensitivity and moderate for low specificity and precision, through advanced search: PubMed, Embase, Cochrane; BVS Portal (main). Other sources, websites, metasearch tools are also part of the strategy. Below is a demonstration of the design of the search strategies of the four fundamental bases.

.

- 1. **PUBMED**

**#1** "Head-Down Tilt"[Mesh] OR (Tilt, Head-Down) OR (Trendelenburg Position) OR (Position, Trendelenburg)

**328 SCIENTIFIC ARTICLES**

**#2** "Surgical Procedures, Operative"[Mesh] OR Operative Procedure* OR Procedure*, Operative OR Surgical Procedure, Operative OR Operative Surgical Procedures OR Procedure*, Operative Surgical OR Surgical Procedures OR Procedure*, Surgical OR Surgical Procedure OR Operative Surgical Procedure OR Surgery, Ghost OR Ghost Surgery

**159.753 SCIENTIFIC ARTICLES**

**#1** Head-Down Tilt"[Mesh] OR (Tilt, Head-Down) OR (Trendelenburg Position) OR (Position, Trendelenburg)

**AND**

**#2** "Surgical Procedures, Operative"[Mesh] OR Operative Procedure* OR Procedure*, Operative OR Surgical Procedure, Operative OR Operative Surgical Procedures OR Procedure*, Operative Surgical OR Surgical Procedures OR Procedure*, Surgical OR Surgical Procedure OR Operative Surgical Procedure OR Surgery, Ghost OR Ghost Surgery

**171 SCIENTIFIC ARTICLES RECOVERED**

**#1** Head-Down Tilt"[Mesh] OR (Tilt, Head-Down) OR (Trendelenburg Position) OR (Position, Trendelenburg)

**#2** "Surgery"[Mesh] OR (Surgery,General) OR (Surgical Procedures, Operative)

**#3** "Intracranial Pressure"[Mesh] OR (Intracranial Hypertension) OR Papilledema

**12 SCIENTIFIC ARTICLES RECOVERED (DUPLICITY) -** **STRATEGY ABOVE**

- 1. **COCHRANE**

**#1** MeSH descriptor: [Head-Down Tilt] explode all trees

**242 SCIENTIFIC ARTICLES**

**#2** MeSH descriptor: [Surgical Procedures, Operative] explode all trees

**124.563 SCIENTIFIC ARTICLES**

**#1** MeSH descriptor: [Head-Down Tilt] explode all trees

**AND**

**#2** MeSH descriptor: [Surgical Procedures, Operative] explode all trees

**101 SCIENTIFIC ARTICLES RECOVERED (100 TRAILS + 1 SR)**

- 1. **EMBASE**

**#1** 'surgery'/exp OR (diagnosis, surgical) OR (diagnostic techniques, surgical) OR operation OR (operation care) OR (operative intervention) OR (operative repair) OR (operative restoration) OR (operative surgery) OR (operative

surgical procedure) OR (operative surgical procedures) OR (operative

treatment) OR (research surgery) OR resection OR (specialties, surgical) OR (surgery, operative) OR (surgical care) OR (surgical correction) OR (surgical diagnosis) OR (surgical diagnostic techniques) OR (surgical exposure) OR (surgical intervention) OR (surgical management) OR (surgical operation) OR (surgical practice) OR (surgical procedures, operative) OR (surgical repair) OR (surgical research) OR (surgical restoration) OR (surgical service) OR (surgical speciality) OR (surgical specialties) OR (surgical specialty) OR (surgical therapy) OR (surgical treatment)

**6.593.424 SCIENTIFIC ARTICLES**

**#2** 'head-down tilt'/exp

**260 SCIENTIFIC ARTICLES RECOVERED**

**#1** ‘surgery’/exp OR (diagnosis, surgical) OR (diagnostic techniques, surgical) OR operation OR (operation care) OR (operative intervention) OR (operative repair) OR (operative restoration) OR (operative surgery) OR (operative

surgical procedure) OR (operative surgical procedures) OR (operative

treatment) OR (research surgery) OR resection OR (specialties, surgical) OR (surgery, operative) OR (surgical care) OR (surgical correction) OR (surgical diagnosis) OR (surgical diagnostic techniques) OR (surgical exposure) OR (surgical intervention) OR (surgical management) OR (surgical operation) OR (surgical practice) OR (surgical procedures, operative) OR (surgical repair) OR

(surgical research) OR (surgical restoration) OR (surgical service) OR (surgical speciality) OR (surgical specialties) OR (surgical specialty) OR (surgical therapy) OR (surgical treatment)

**AND**

**#2** ‘head-down tilt’/exp

**80 SCIENTIFIC ARTICLES RECOVERED**

- 1. **ARTIGOS BVS**

**#1** MH: "Decúbito Inclinado com Rebaixamento da Cabeça" OR (Head-Down Tilt) OR (Inclinación de Cabeza) OR (Decúbito Inclinado com Cabeça para Baixo) OR (Posição de Trendelenburg) OR (Position, Trendelenburg) OR (Tilt, Head-Down) OR (Trendelenburg Position) OR (Posición de Trendelenburg) OR MH:G11.427.695.300$

**4.312 SCIENTIFIC ARTICLES**

**#2** MH:"Cirurgia Geral" OR (General Surgery) OR (Cirugía General) OR Cirurgia OR MH:"Procedimentos Cirúrgicos Operatórios" OR (Surgical Procedures, Operative) OR (Procedimientos Quirúrgicos Operativos) OR (Intervenção* Cirúrgica) OR (Operação* Cirúrgica) OR (Procedimento* Cirúrgico*) OR (Procedimento Cirúrgico Operatório) OR (Procedimento* Operatório*) OR MH:H02.403.810.300$ OR MH:E04$ OR MH:VS3.003.001.006.002$

**4.063.152 SCIENTIFIC ARTICLES**

**#1** MH: "Decúbito Inclinado com Rebaixamento da Cabeça" OR (Head-Down Tilt) OR (Inclinación de Cabeza) OR (Decúbito Inclinado com Cabeça para Baixo) OR (Posição de Trendelenburg) OR (Position, Trendelenburg) OR (Tilt, Head-Down) OR (Trendelenburg Position) OR (Posición de Trendelenburg) OR MH:G11.427.695.300$

**AND**

**#2** MH:"Cirurgia Geral" OR (General Surgery) OR (Cirugía General) OR Cirurgia OR MH:"Procedimentos Cirúrgicos Operatórios" OR (Surgical Procedures, Operative) OR (Procedimientos Quirúrgicos Operativos) OR (Intervenção* Cirúrgica) OR (Operação* Cirúrgica) OR (Procedimento* Cirúrgico*) OR (Procedimento Cirúrgico Operatório) OR (Procedimento* Operatório*) OR MH:H02.403.810.300$ OR MH:E04$ OR MH:VS3.003.001.006.002$

**1.347 SCIENTIFIC ARTICLES**

**APPLICATION OF FILTERS**

Removed MEDLINE, leaving everything related to laparoscopy and robotics, pneumoperitoneum, per and postoperative complications.

**12 SCIENTIFIC ARTICLES RECOVERED**

- 1. **TRIPDATABASE**

**#1** head-down tilt AND surgical procedures, operative.

**19 RECOVERED MATERIALS**

**2 SCIENTIFIC ARTICLES RECOVERED**

- 1. **CLINICALTRAIL.GOV**

**#1** head-down tilt AND surgical procedures, operative.

**7 RECOVERED MATERIALS**

**1 USEFUL STUDY NOT YET PUBLISHED**

- 1. **UNIVERSITY OF YORK**

**#1** head-down tilt AND surgical procedures, operative.

**ZERO TRAILS**

- 1. **EPISTEMONIKOS**

**#1** (Head-Down Tilt) OR (Tilt, Head-Down) OR (Trendelenburg Position) OR (Position, Trendelenburg)

**152.236 SCIENTIFIC ARTICLES**

**#2** surgery OR (diagnosis, surgical) OR (diagnostic techniques, surgical) OR operation OR (operation care) OR (operative intervention) OR (operative repair) OR (operative restoration) OR (operative surgery) OR (operative surgical procedure) OR (operative surgical procedures) OR (operative treatment) OR (research surgery) OR resection OR (specialties, surgical) OR (surgery, operative) OR (surgical care) OR (surgical correction) OR (surgical diagnosis) OR (surgical diagnostic techniques) OR (surgical exposure) OR (surgical intervention) OR (surgical management) OR

(surgical operation) OR (surgical practice) OR (surgical procedures,

operative) OR (surgical repair) OR (surgical research) OR (surgical restoration) OR (surgical service) OR (surgical speciality) OR (surgical specialties) OR (surgical specialty) OR (surgical therapy) OR (surgical treatment)

**#3** (ophthalmic nerve) OR (nerve, ophthalmic) OR (nervus ophthalmicus) OR optic disk OR (discus nervus optici) OR (eye disk) OR (optic disc) OR (optic papilla)

**#4** (Intracranial Pressure) OR (Intracranial Hypertension) OR (Pressure, Intracranial) OR (Pressures, Intracranial) OR (ICP, Elevated (Intracranial Pressure)) OR (ICP (Intracranial Pressure) Elevation) OR ICP (Intracranial Pressure) Increase) OR (Elevated ICP (Intracranial Pressure)) OR (Pressure, Elevated Intracranial) OR (Intracranial Pressure, Elevated) OR (Pressure Increase, Intracranial) OR (Intracranial Pressure Increase) OR (Elevated Intracranial Pressure) OR (Hypertension, Intracranial) OR (Subarachnoid Pressures) OR (Pressure, Intracranial) OR (Pressure, Subarachnoid) OR (Subarachnoid Pressure) OR (Intracranial Pressures) OR (Pressures, Subarachnoid) OR (Pressures, Intracranial) OR (Pressures, Intracerebral) OR (Intracerebral Pressures) OR (Intracerebral Pressure) OR (Pressure, Intracerebral).

**#5** = #1 AND #2

(Head-Down Tilt) OR (Tilt, Head-Down) OR (Trendelenburg Position) OR (Position, Trendelenburg) AND surgery OR (diagnosis, surgical) OR (diagnostic techniques, surgical) OR operation OR (operation care) OR (operative intervention) OR (operative repair) OR (operative restoration) OR (operative surgery) OR (operative surgical procedure) OR (operative surgical procedures) OR (operative treatment) OR (research surgery) OR resection OR (specialties, surgical) OR (surgery, operative) OR (surgical care) OR (surgical correction) OR (surgical diagnosis) OR (surgical diagnostic techniques) OR (surgical exposure) OR (surgical intervention) OR (surgical management) OR (surgical operation) OR (surgical practice) OR (surgical procedures, operative) OR (surgical repair) OR (surgical research) OR (surgical restoration) OR (surgical service) OR (surgical speciality) OR (surgical specialties) OR (surgical specialty) OR (surgical therapy) OR (surgical treatment)

**12.960 SCIENTIFIC ARTICLES**

**#6** = #5 AND #3 AND #4

**(**Head-Down Tilt) OR (Tilt, Head-Down) OR (Trendelenburg Position) OR (Position, Trendelenburg) AND surgery OR (diagnosis, surgical) OR (diagnostic techniques, surgical) OR operation OR (operation care) OR (operative intervention) OR (operative repair) OR (operative restoration) OR (operative surgery) OR (operative surgical procedure) OR (operative surgical procedures) OR (operative treatment) OR (research surgery) OR

resection OR (specialties**,** surgical) OR (surgery, operative) OR (surgical care) OR (surgical correction) OR (surgical diagnosis) OR (surgical diagnostic techniques) OR (surgical exposure) OR (surgical intervention) OR (surgical management) OR (surgical operation) OR (surgical practice) OR (surgical procedures, operative) OR (surgical repair) OR (surgical research) OR (surgical restoration) OR (surgical service) OR (surgical speciality) OR (surgical specialties) OR (surgical specialty) OR (surgical therapy) OR (surgical treatment) AND (ophthalmic nerve) OR (nerve, ophthalmic) OR (nervus ophthalmicus) OR optic disk OR (discus nervus optici) OR (eye disk) OR (optic disc) OR (optic papilla) AND (Intracranial Pressure) OR (Intracranial Hypertension**)** OR (Pressure, Intracranial) OR (Pressures, Intracranial) OR (ICP, Elevated (Intracranial Pressure)) OR (ICP (Intracranial Pressure) Elevation) OR ICP (Intracranial Pressure) Increase) OR (Elevated ICP (Intracranial Pressure)) OR (Pressure, Elevated Intracranial) OR (Intracranial Pressure, Elevated) OR (Pressure Increase, Intracranial) OR (Intracranial Pressure Increase) OR (Elevated Intracranial Pressure) OR (Hypertension, Intracranial) OR (Subarachnoid Pressures) OR (Pressure, Intracranial) OR (Pressure, Subarachnoid) OR (Subarachnoid Pressure) OR (Intracranial Pressures) OR (Pressures, Subarachnoid) OR (Pressures, Intracranial) OR (Pressures, Intracerebral) OR (Intracerebral Pressures) OR (Intracerebral Pressure) OR (Pressure, Intracerebral)

**7 SCIENTIFIC ARTICLES**

**1 SCIENTIFIC ARTICLES RECOVERED**

- 1. **TRIPDATABASE**

**#1** (Head-Down Tilt) OR (Tilt, Head-Down) OR (Trendelenburg Position) OR (Position, Trendelenburg)

**1.286 SCIENTIFIC ARTICLES**

**#2** surgery OR (diagnosis, surgical) OR (diagnostic techniques, surgical) OR operation OR (operation care) OR (operative intervention) OR (operative repair) OR (operative restoration) OR (operative surgery) OR (operative surgical procedure) OR (operative surgical procedures) OR (operative treatment) OR (research surgery) OR resection OR (specialties, surgical) OR (surgery, operative) OR (surgical care) OR (surgical correction) OR (surgical diagnosis) OR (surgical diagnostic techniques) OR (surgical exposure) OR (surgical intervention) OR (surgical management) OR (surgical operation) OR (surgical practice) OR (surgical procedures,

operative) OR (surgical repair) OR (surgical research) OR (surgical restoration) OR (surgical service) OR (surgical speciality) OR (surgical specialties) OR (surgical specialty) OR (surgical therapy) OR (surgical treatment)

**#3** (ophthalmic nerve) OR (nerve, ophthalmic) OR (nervus ophthalmicus) OR optic disk OR (discus nervus optici) OR (eye disk) OR (optic disc) OR (optic papilla)

**#4** (Intracranial Pressure) OR (Intracranial Hypertension) OR (Pressure, Intracranial) OR (Pressures, Intracranial) OR (ICP, Elevated (Intracranial Pressure)) OR (ICP (Intracranial Pressure) Elevation) OR ICP (Intracranial Pressure) Increase) OR (Elevated ICP (Intracranial Pressure)) OR (Pressure, Elevated Intracranial) OR (Intracranial Pressure, Elevated) OR (Pressure Increase, Intracranial) OR (Intracranial Pressure Increase) OR (Elevated Intracranial Pressure) OR (Hypertension, Intracranial) OR (Subarachnoid Pressures) OR (Pressure, Intracranial) OR (Pressure, Subarachnoid) OR (Subarachnoid Pressure) OR (Intracranial Pressures) OR (Pressures, Subarachnoid) OR (Pressures, Intracranial) OR (Pressures, Intracerebral) OR (Intracerebral Pressures) OR (Intracerebral Pressure) OR (Pressure, Intracerebral).

**#5 = #1 AND #2**

(Head-Down Tilt) OR (Tilt, Head-Down) OR (Trendelenburg Position) OR (Position, Trendelenburg) AND surgery OR (diagnosis, surgical) OR (diagnostic techniques, surgical) OR operation OR (operation care) OR (operative intervention) OR (operative repair) OR (operative restoration) OR (operative surgery) OR (operative surgical procedure) OR (operative surgical procedures) OR (operative treatment) OR (research surgery) OR resection OR (specialties, surgical) OR (surgery, operative) OR (surgical care) OR (surgical correction) OR (surgical diagnosis) OR (surgical diagnostic techniques) OR (surgical exposure) OR (surgical intervention) OR (surgical management) OR (surgical operation) OR (surgical practice) OR (surgical procedures, operative) OR (surgical repair) OR (surgical research) OR (surgical restoration) OR (surgical service) OR (surgical speciality) OR (surgical specialties) OR (surgical specialty) OR (surgical therapy) OR (surgical treatment)

**1.286 SCIENTIFIC ARTICLES**

**#6 = #5 AND #3 AND #4**

(Head-Down Tilt) OR (Tilt, Head-Down) OR (Trendelenburg Position) OR (Position, Trendelenburg) AND surgery OR (diagnosis, surgical) OR (diagnostic techniques, surgical) OR operation OR (operation care) OR (operative intervention) OR (operative repair) OR (operative restoration) OR (operative surgery) OR (operative surgical procedure) OR (operative surgical procedures) OR (operative treatment) OR (research surgery) OR resection OR (specialties, surgical) OR (surgery, operative) OR (surgical care) OR (surgical correction) OR (surgical diagnosis) OR (surgical diagnostic techniques) OR (surgical exposure) OR (surgical intervention) OR (surgical management) OR (surgical operation) OR (surgical practice) OR (surgical procedures, operative) OR (surgical repair) OR (surgical research) OR (surgical restoration) OR (surgical service) OR (surgical speciality) OR (surgical specialties) OR (surgical specialty) OR (surgical therapy) OR (surgical treatment) AND (ophthalmic nerve) OR (nerve, ophthalmic) OR (nervus ophthalmicus) OR optic disk OR (discus nervus optici) OR (eye disk) OR (optic disc) OR (optic papilla) AND (Intracranial Pressure) OR (Intracranial Hypertension) OR (Pressure, Intracranial) OR (Pressures, Intracranial) OR (ICP, Elevated (Intracranial Pressure)) OR (ICP (Intracranial Pressure) Elevation) OR ICP (Intracranial Pressure) Increase) OR (Elevated ICP (Intracranial Pressure)) OR (Pressure, Elevated Intracranial) OR (Intracranial Pressure, Elevated) OR (Pressure Increase, Intracranial) OR (Intracranial Pressure Increase) OR (Elevated Intracranial Pressure) OR (Hypertension, Intracranial) OR (Subarachnoid Pressures) OR (Pressure, Intracranial) OR (Pressure, Subarachnoid) OR (Subarachnoid Pressure) OR (Intracranial Pressures) OR (Pressures, Subarachnoid) OR (Pressures, Intracranial) OR (Pressures, Intracerebral) OR (Intracerebral Pressures) OR (Intracerebral Pressure) OR (Pressure, Intracerebral)

**7 SCIENTIFIC ARTICLES**

**ZERO USEFUL ARTICLE**

- 1. **WORDWIDESCIENCE.ORG**

#1 (Head-Down Tilt) OR (Tilt, Head-Down) OR (Trendelenburg Position) OR (Position, Trendelenburg)

**1.139 SCIENTIFIC ARTICLES**

**#2** surgery OR (diagnosis, surgical) OR (diagnostic techniques, surgical) OR operation OR (operation care) OR (operative intervention) OR (operative repair) OR (operative restoration) OR (operative surgery) OR (operative surgical procedure) OR (operative surgical procedures) OR (operative treatment) OR (research surgery) OR resection OR (specialties, surgical) OR (surgery, operative) OR (surgical care) OR (surgical correction) OR (surgical diagnosis) OR (surgical diagnostic techniques) OR (surgical exposure) OR (surgical intervention) OR (surgical management) OR

(surgical operation) OR (surgical practice) OR (surgical procedures,

operative) OR (surgical repair) OR (surgical research) OR (surgical restoration) OR (surgical service) OR (surgical speciality) OR (surgical specialties) OR (surgical specialty) OR (surgical therapy) OR (surgical treatment).

**#3** (ophthalmic nerve) OR (nerve, ophthalmic) OR (nervus ophthalmicus) OR optic disk OR (discus nervus optici) OR (eye disk) OR (optic disc) OR (optic papilla).

**#4** (Intracranial Pressure) OR (Intracranial Hypertension) OR (Pressure, Intracranial) OR (Pressures, Intracranial) OR (ICP, Elevated (Intracranial Pressure)) OR (ICP (Intracranial Pressure) Elevation) OR ICP (Intracranial Pressure) Increase) OR (Elevated ICP (Intracranial Pressure)) OR (Pressure, Elevated Intracranial) OR (Intracranial Pressure, Elevated) OR (Pressure Increase, Intracranial) OR (Intracranial Pressure Increase) OR (Elevated Intracranial Pressure) OR (Hypertension, Intracranial) OR (Subarachnoid Pressures) OR (Pressure, Intracranial) OR (Pressure, Subarachnoid) OR (Subarachnoid Pressure) OR (Intracranial Pressures) OR (Pressures, Subarachnoid) OR (Pressures, Intracranial) OR (Pressures, Intracerebral) OR (Intracerebral Pressures) OR (Intracerebral Pressure) OR (Pressure, Intracerebral).

**#5 = #1 AND #2**

(Head-Down Tilt) OR (Tilt, Head-Down) OR (Trendelenburg Position) OR (Position, Trendelenburg) AND surgery OR (diagnosis, surgical) OR (diagnostic techniques, surgical) OR operation OR (operation care) OR (operative intervention) OR (operative repair) OR (operative restoration) OR (operative surgery) OR (operative surgical procedure**)** OR (operative surgical procedures) OR (operative treatment) OR (research surgery) OR resection OR (specialties, surgical) OR (surgery, operative) OR (surgical care) OR (surgical correction) OR (surgical diagnosis) OR (surgical diagnostic techniques) OR (surgical exposure) OR (surgical intervention)

OR (surgical management) OR (surgical operation) OR (surgical practice) OR (surgical procedures, operative) OR (surgical repair) OR (surgical research) OR (surgical restoration) OR (surgical service) OR (surgical speciality) OR (surgical specialties) OR (surgical specialty) OR (surgical therapy) OR (surgical treatment)

**466 SCIENTIFIC ARTICLES**

**#6 = #5 AND #3 AND #4**

(Head-Down Tilt) OR (Tilt, Head-Down) OR (Trendelenburg Position) OR (Position, Trendelenburg) AND surgery OR (diagnosis, surgical) OR (diagnostic techniques, surgical) OR operation OR (operation care) OR (operative intervention) OR (operative repair) OR (operative restoration) OR (operative surgery) OR (operative surgical procedure) OR (operative surgical procedures) OR (operative treatment) OR (research surgery) OR resection OR (specialties, surgical) OR (surgery, operative) OR (surgical care) OR (surgical correction) OR (surgical diagnosis) OR (surgical diagnostic techniques) OR (surgical exposure) OR (surgical intervention) OR (surgical management) OR (surgical operation) OR (surgical practice) OR (surgical procedures, operative) OR (surgical repair) OR (surgical research) OR (surgical restoration) OR (surgical service) OR (surgical speciality) OR (surgical specialties) OR (surgical specialty) OR (surgical therapy) OR (surgical treatment) AND (ophthalmic nerve) OR (nerve, ophthalmic) OR (nervus ophthalmicus) OR optic disk OR (discus nervus optici) OR (eye disk) OR (optic disc) OR (optic papilla) AND (Intracranial Pressure) OR (Intracranial Hypertension) OR (Pressure, Intracranial) OR (Pressures, Intracranial) OR (ICP, Elevated (Intracranial Pressure)) OR (ICP (Intracranial Pressure) Elevation) OR ICP (Intracranial Pressure) Increase) OR (Elevated ICP (Intracranial Pressure)) OR (Pressure, Elevated Intracranial) OR (Intracranial Pressure, Elevated) OR (Pressure Increase, Intracranial) OR (Intracranial Pressure Increase) OR (Elevated Intracranial Pressure) OR (Hypertension, Intracranial) OR (Subarachnoid Pressures) OR (Pressure, Intracranial) OR (Pressure, Subarachnoid) OR (Subarachnoid Pressure) OR (Intracranial Pressures) OR (Pressures, Subarachnoid) OR (Pressures, Intracranial) OR (Pressures, Intracerebral) OR (Intracerebral Pressures) OR (Intracerebral Pressure) OR (Pressure, Intracerebral)

**# 7** (Head-Down Tilt) OR (Tilt, Head-Down) OR (Trendelenburg Position) OR (Position, Trendelenburg) AND surgery OR (diagnosis, surgical) OR (diagnostic techniques, surgical) OR operation OR (operation care) OR

(operative intervention) OR (operative repair) OR (operative restoration) OR (operative surgery) OR (operative surgical procedure) AND (ophthalmic nerve) OR (nerve, ophthalmic) OR (nervus ophthalmicus) OR optic disk OR (discus nervus optici) OR (eye disk) OR (optic disc) OR (optic papilla)

**236 SCIENTIFIC ARTICLES**

**#8** Pneumoperitoneum

**#9** (Head-Down Tilt) OR (Tilt, Head-Down) OR (Trendelenburg Position) OR (Position, Trendelenburg) AND surgery OR (diagnosis, surgical) OR (diagnostic techniques, surgical) OR operation OR (operation care) OR (operative intervention) OR (operative repair) OR (operative restoration) OR (operative surgery) OR (operative surgical procedure) AND (ophthalmic nerve) OR (nerve, ophthalmic) OR (nervus ophthalmicus) OR optic disk OR (discus nervus optici) OR (eye disk) OR (optic disc) OR (optic papilla) AND Pneumoperitoneum

**369 SCIENTIFIC ARTICLES**

**#10** Laparoscopy OR Laparoscopies OR Celioscopy OR Celioscopies OR Peritoneoscopy OR Peritoneoscopies OR (Surgical Procedures, Laparoscopic) OR (Laparoscopic Surgical Procedure) OR (Procedure*, Laparoscopic Surgical) OR (Surgery, Laparoscopic) OR (Laparoscopic Surgical Procedures) OR (Laparoscopic* Surgery) OR (Surgeries, Laparoscopic) OR (Laparoscopic* Assisted Surgery) OR (Surgery*, Laparoscopic Assisted) OR (Surgical Procedure, Laparoscopic)

**#11** (Robotic Surgical Procedures) OR Procedure*, Robotic Surgical OR (Robotic Surgical Procedure) OR (Surgical Procedure, Robotic) OR (Robot Surgery*) OR (Surgery, Robot) OR (Robot-Assisted Surgery*) OR (Robot Assisted Surgery) OR (Surgery, Robot-Assisted) OR (Robot-Enhanced Procedure*) OR (Procedure, Robot-Enhanced) OR (Robot Enhanced Procedures) OR (Surgical Procedures, Robotic) OR (Robotic-Assisted Surgery) OR (Robotic Assisted Surgery) OR Robotic-Assisted Surgeries OR Surgery, Robotic-Assisted OR Robot-Enhanced Surgery OR Robot Enhanced Surgery OR Robot-Enhanced Surgeries OR Surgery, Robot-

Enhanced

**#12** (Head-Down Tilt) OR (Tilt, Head-Down) OR (Trendelenburg Position) OR (Position, Trendelenburg) AND surgery OR (diagnosis, surgical) OR (diagnostic techniques, surgical) OR operation OR (operation care) OR (operative intervention) OR (operative repair) OR (operative restoration)

OR (operative surgery) OR (operative surgical procedure) AND (ophthalmic nerve) OR (nerve, ophthalmic) OR (nervus ophthalmicus) OR optic disk OR (discus nervus optici) OR (eye disk) OR (optic disc) OR (optic papilla) AND Laparoscopy OR Laparoscopies OR Celioscopy OR Celioscopies OR Peritoneoscopy OR Peritoneoscopies OR (Surgical Procedures, Laparoscopic) OR (Laparoscopic Surgical Procedure) OR (Procedure*, Laparoscopic Surgical) OR (Surgery, Laparoscopic) OR (Laparoscopic Surgical Procedures) OR (Laparoscopic* Surgery) OR (Surgeries, Laparoscopic) OR (Laparoscopic* Assisted Surgery) OR (Surgery*, Laparoscopic Assisted) OR (Surgical Procedure, Laparoscopic) AND (Robotic Surgical Procedures) OR Procedure*, Robotic Surgical OR (Robotic Surgical Procedure) OR (Surgical Procedure, Robotic) OR (Robot Surgery*) OR (Surgery, Robot) OR (Robot- Assisted Surgery*) OR (Robot Assisted Surgery) OR (Surgery, Robot-Assisted) OR (Robot-Enhanced Procedure*) OR (Procedure, Robot-Enhanced) OR (Robot Enhanced Procedures) OR (Surgical Procedures, Robotic) OR (Robotic- Assisted Surgery) OR (Robotic Assisted Surgery) OR Robotic- Assisted Surgeries OR Surgery, Robotic-Assisted OR Robot-Enhanced Surgery OR Robot Enhanced Surgery OR Robot-Enhanced Surgeries OR Surgery, Robot- Enhanced

**89 SCIENTIFIC ARTICLES**

**ZERO USEFUL ARTICLE**

**#13** head-down tilt AND surgical procedures, operative AND (ophthalmic nerve) OR (nerve, ophthalmic) OR (nervus ophthalmicus) OR optic disk OR (discus nervus optici) OR (eye disk) OR (optic disc) OR (optic papila) AND Pneumoperitoneum

**10 SCIENTIFIC ARTICLES RECOVERED**

- 1. **ARTIGOS BMJ EVIDENCE-BASED MEDICINE**

**#1** head-down tilt AND surgical procedures, operative AND (ophthalmic nerve) OR (nerve, ophthalmic) OR (nervus ophthalmicus) OR optic disk OR (discus nervus optici) OR (eye disk) OR (optic disc) OR (optic papila) AND Pneumoperitoneum

**4 SCIENTIFIC ARTICLES**

**ZERO USEFUL ARTICLE**

**#2** (Head-Down Tilt) OR (Tilt, Head-Down) OR (Trendelenburg Position) OR (Position, Trendelenburg)

**1.135 SCIENTIFIC ARTICLES**

**#3** (Head-Down Tilt) OR (Tilt, Head-Down) OR (Trendelenburg Position) AND Pneumoperitoneum

**ZERO SCIENTIFIC ARTICLES**

**#4** (Head-Down Tilt) OR (Tilt, Head-Down) OR (Trendelenburg Position) AND surgery OR Surgical Procedures, Operative

**200 SCIENTIFIC ARTICLES RECOVERED**

- 1. **Qinsigth**

**#1** (Head-Down Tilt) OR (Tilt, Head-Down) OR (Trendelenburg Position) AND surgery OR Surgical Procedures, Operative

**#2** Trendelenburg Position AND Pneumoperitoneum

**ZERO SCIENTIFIC ARTICLES RECOVERED**

- 1. **Oasisbr e ARTIGOS medRxiv**

**#1** (Head-Down Tilt) OR (Tilt, Head-Down) OR (Trendelenburg Position) AND surgery OR (Surgical Procedures, Operative)

**3.473 SCIENTIFIC ARTICLES**

**#2** (Head-Down Tilt) OR (Trendelenburg Position) AND surgery OR (Surgical Procedures, Operative) AND Pneumoperitoneum

**2 SCIENTIFIC ARTICLES**

**#3** (head-down tilt) AND (surgical procedures, operative) AND (ophthalmic nerve) OR (nerve, ophthalmic)

**3 SCIENTIFIC ARTICLES**

**#4** (Head-Down Tilt) OR (Trendelenburg Position) AND surgery OR (Surgical Procedures, Operative) AND (Intracranial presurre)

**100 SCIENTIFIC ARTICLES**

**1 USEFUL ARTICLE**

- 1. **Scielo Preprint**

**770 SCIENTIFIC ARTICLES ZERO USEFUL ARTICLE**

- 1. **HANSEARCH**

**3 USEFUL ARTICLE**

**Appendix 2**

**STATISTIC**

**1. Method**

*1.1 Data extraction and management*

Data extraction and management were performed independently by two authors (VTC and NCJ) using an extraction form in Excel software. Data were extracted on methods (study design and definition), identifying data (funding source, country and author details such as name, institution, email and address), data on participant characteristics (number of participants randomized, number of participants analyzed and number lost to follow-up with reasons given; baseline data characteristics; inclusion criteria), interventions (number of participants within each intervention group and intervention description), outcome measures (outcome type, reporting, range, unit of measurement, direction, and observations), study design characteristics (and risk of bias assessment) and any other relevant information.

Two authors (VTC and NCJ) independently extracted outcome data from the included studies. For the continuous outcome included, the means, standard deviations and number of participants included in each intervention group were extracted.

The difference between the post-intervention time point and the baseline was prioritized in data extraction or transformation due to imbalances in the baseline time points of some studies. Data extraction was based on intention-to-treat analysis, when possible.

In case of disagreements, conflicts were assessed until consensus was reached and, after this phase, one review author exported the data to the Review Manager 5 software to avoid inconsistencies.

*1.2 Assessment of risk of bias in included studies*

Two review authors (VTC and NCJ) independently assessed the risk of bias of each included study using version 2 of the Cochrane ‘Risk of Bias’ tool (RoB2) in accordance with the recommendations in Chapter 8 of the Cochrane Handbook for Systematic Reviews of Interventions version 6.3.1. All disagreements were resolved by consensus, using the following definitions in assessing risk of bias: bias due to the randomization process; bias due to deviations from the planned interventions; bias due to missing outcome data; bias in the measurement of the outcome; bias in the selection of reported outcomes.

The assessment focused on the effect of assignment to the interventions at baseline (the ‘intention-to-treat effect’). For the flagging questions within each domain for each outcome, one of five possible responses was provided in the RoB2 tool (‘Yes’, ‘Probably yes’, ‘No’, ‘Probably not’ and ‘No information’), judging as ‘Low risk of bias’, ‘Some concerns’ or ‘High risk of bias’ according to the algorithm result.

*1.3 Treatment effect measures*

For continuous data, mean, Standard Deviation (SD) and number of participants in each intervention group were extracted from the included trials. When trials reported any other measure of dispersion (e.g., confidence interval or standard error), the SD was calculated according to the instructions in chapter 6 of the Cochrane Handbook for Systematic Reviews of Interventions version 6.3.2. Data were summarized using meta-analysis by the inverse variance method and random-effects model, since one of the studies presented results separated by eye, which were subsequently pooled also following the instructions in chapter 6 of the Cochrane Handbook for Systematic Reviews of Interventions version 6.3.2.

*1.4 Unit of analysis*

The unit of analysis considered was each individual (the randomization of the studies was used based on each individual participant).

When several time points were reported in the same study, the data related to the longest follow-up of the surgical time in which the patient underwent pneumoperitoneum were included (except for the study by Sujata et al in which the data extracted was related to the highest value found due to the availability of data with the aim of evaluating the worst-case scenario).

*1.5 Lost data management*

Outcome data were extracted, when available, using intention-to-treat analysis (all participants randomized) or modified Intention-To-Treat (ITT) analysis (assumptions decided by study authors). If data were not available, only the analysis with reported data was performed. In this case, the impact of including these trials on the overall assessment of the meta-analyses would be explored by a sensitivity analysis.

*1.6 Heterogeneity assessment*

Potential clinical heterogeneity was assessed considering participants, interventions, outcomes, and study characteristics for the included trials.

Statistical heterogeneity was visually inspected in forest plots, and the Chi2 test was used considering a threshold of p < 0.1 as an indicator of the presence of heterogeneity. In addition, the I^2^ statistic was used to describe the proportion of variation in effect estimates that was due to between-study variability rather than sampling error.

The I^2^ statistic was used following the recommendations of chapter 10 of the Cochrane Handbook for Systematic Reviews of Interventions version 6.3:3

• 0 a 40%: may not be important;

• 30% a 60%: may represent moderate heterogeneity;

• 50% a 74%: may represent substantial heterogeneity;

• 75% a 100%: considerable heterogeneity.

*1.7 Assessment of reporting biases*

Investigation of publication bias was planned using funnel plots if at least 10 studies were included in the meta-analysis. However, this assessment was not possible due to fewer than 10 trials being included in the meta-analysis.

Trial protocol records were checked when available to assess outcome reporting bias.

*1.8 Data synthesis*

Meta-analysis was performed if more than one trial was included for the comparison of interest. Meta-analysis was performed by pooling included studies only if participants, interventions, and outcomes were similar enough to pool.

Statistical analyses were performed according to the recommendations outlined in Chapter 10 of the Cochrane Handbook for Systematic Reviews of Interventions.[3] Continuous outcomes were summarized using MD in a meta-analysis using the inverse variance method and random-effects model. Data were analyzed using Review Manager 5 software.

1.8.1 Table of key findings, assessment of certainty of evidence

A Table of the main findings for the outcome was created based on the comparison of interest.

Regarding the assessment of the certainty of the evidence, two authors (VTC and NCJ) used the approach proposed by the GRADE Working Group and the recommendations of chapter 14 of the Cochrane Handbook for Systematic Reviews of Interventions version 6.3.4. The GRADE pro GDT software was used to analyze the overall certainty of the evidence for each outcome, and any disagreement was resolved by consensus. The certainty of the evidence was downgraded by one level for serious limitations or two levels for very serious limitations based on defined criteria (study limitations, inconsistency, indirect evidence, imprecision of estimates, and presence of publication bias). In the case of imprecision, the study could be downgraded up to three levels in relation to certainty. In the end, each outcome was categorized into four levels (high certainty, moderate certainty, low certainty, and very low certainty). Each decision to downgrade the certainty of the evidence was justified in footnotes.

*1.9 Subgroup analysis and investigation of heterogeneity*

A subgroup analysis for surgery time was planned, but due to the low importance of the heterogeneity found, this investigation was not necessary.

*1.10 Sensitivity analysis*

A sensitivity analysis excluding studies with high risk of bias was planned, however it was not necessary as no studies were assessed as having high risk of bias overall.

**2. Results**

*2.1 Description of studies*

The characteristics of each included study were described in the ‘Characteristics of included studies’ table (Table 1 in Annex 1).

*2.2 Risk of bias in included studies*

Risk of bias assessments for each included study were expressed directly in the meta-analyses, and decisions between different types of bias were shown in Figure 1 below.


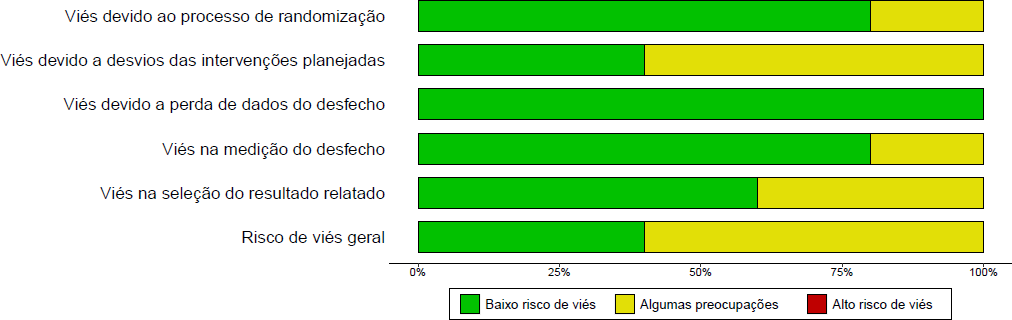

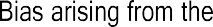

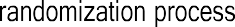

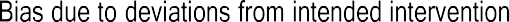

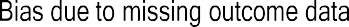

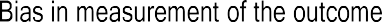

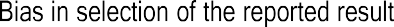

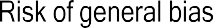


High risk of bias

Some concerns

low risk of bias

**Figura 1.** Risk of general bias of studies included in the systematic review.

2.2.1 Randomization process

Four studies (80%) reported an adequate method of random sequence generation, such as a computer-generated draw or random number table, and ensured allocation concealment by the use of opaque envelopes, sealed and sealed by independent investigators, allowing for balancing between group characteristics. Only one study (20%) raised some concerns about the randomization process related to random sequence generation or allocation concealment.

2.2.2 Deviations from planned interventions

Two studies (40%) described interventions that were indistinguishable in method of delivery, ensuring double-masking or analyzing in a way that ensured attribution effect (intention to treat). The other three studies (60%) reported insufficient information to judge blinding or did not describe adequate methods of analysis to avoid risk of bias from deviation from planned interventions.

2.2.3 Missing outcome data

All studies reported complete data on randomized participants or presented intention-to-treat analyses appropriate to ensure low risk of bias due to missing outcome data.

2.2.4 Outcome measurement

The outcomes of four studies (80%) were assessed adequately, ensuring low risk of bias, and only one study (20%) did not describe adequate ways of masking the evaluators, leading to some concerns in the judgment of this domain.

2.2.5 Selection of reported result

Three studies (60%) had adequate analysis planning for the outcome through protocol registration, leading to a low risk of bias. Two studies (40%) did not provide sufficient information on analysis planning and outcome result reporting, leading to some concerns.

2.2.6 Overall assessment of risk of bias

Two studies (40%) had low overall risk of bias and three studies (60%) had some concerns for overall risk of bias.

*2.3 Effects of interventions*

2.3.1 Propofol versus sevoflurano

2.3.1.1 Intracranial pressure

Five studies were found including 277 participants for the outcome intracranial pressure (assessed by optic nerve sheath diameter by ultrasound; smaller measurement = better). Propofol probably reduces intracranial pressure slightly when compared with sevoflurane (MD = -0.23 mm, 95% CI -0.37 to -0.10; studies = 5; I^2^ = 23%) (Fig. 2).

**Figure 2** *Forest plot* of the comparison: propofol versus sevoflurane, outcome: Intracranial pressure and GRADE.

Applying the GRADE criteria, moderate-certainty evidence was found that propofol probably slightly reduces intracranial pressure when compared with sevoflurane (downgraded once for imprecision) (Table 2).

**Table 2. Table of main findings** (Propofol versus Sevoflurano).

| Propofol compared to sevoflurane in surgical patients undergoing pneumoperitoneum in the cephaloduck position | | | | | | |
| --- | --- | --- | --- | --- | --- | --- |
| **Patient or population**: Surgical patients undergoing pneumoperitoneum in the cephalodeclination position  **Setting**: Surgery unit  **Intervention**: Propofol  **Comparison**: Sevoflurane | | | | | | |
| Outcomes | **Potential absolute effects* (95% CI)** | | Relative effect (95% CI) | № de participants (studies) | Certainty of evidence (GRADE) | Comments |
|  | **Risk with**  **Sevoflurano** | **Riik with**  **Propofol** |  |  |  |  |
| Intracranial pressure  assessed with: Optic nerve ultrasound (optic nerve sheath diameter);  Follow-up: variation from 0.5 hours to 3 hours | The mean intracranial pressure ranged from 0.26 to 0.9 millimeters | AD 0.23mm less (0.37 less to 0.1 less) | - | 277 (5 RCTs) | ⨁⨁⨁◯ Moderate^a^ | Propofol probably reduces intracranial pressure slightly  .  MCID = 0,25 a 0,5 mm. |
| * **The risk in the intervention group** (and its 95% confidence interval) is based on the assumed risk of the comparator group and the relative effect of the intervention (and its 95% CI).  **CI:** Confidence interval; **AD:** Average difference; **RCT:** Randomized clinical trial; **MCID**: Minimal clinically important difference. | | | | | | |
| **GRADE Working Group grades of evidence**  **High certainty**: High confidence that the true effect is close to the estimate  **Moderate certainty**: Moderate confidence in the effect estimate: The true effect is likely to be close to the effect estimate, but there is a possibility that it is substantially different  **Low certainty**: Limited confidence in the effect estimate: The true effect may be substantially different from the effect estimate  **Very low certainty**: Very little confidence in the effect estimate: The true effect is likely to be substantially different from the effect estimate | | | | | | |

#### Notes:

**a**. Decrease by one level due to imprecision. The 95% CI of the absolute difference ranges from a clinically important benefit to a clinically unimportant benefit, and fewer than 400 participants were involved in the meta-analysis.

**References**

1. Higgins JPT, Savović J, Page MJ, Elbers RG, Sterne JAC. Chapter 8: Assessing risk of bias in a randomized trial. In: Higgins JPT, Thomas J, Chandler J, Cumpston M, Li T, Page MJ, Welch VA (editors). Cochrane Handbook for Systematic Reviews of Interventions version 6.3 (updated February 2022). Cochrane, 2022. Disponível em: www.training.cochrane.org/handbook.

2. Higgins JPT, Li T, Deeks JJ (editors). Chapter 6: Choosing effect measures and computing estimates of effect. In: Higgins JPT, Thomas J, Chandler J, Cumpston M, Li T, Page MJ, Welch VA (editors). Cochrane Handbook for Systematic Reviews of Interventions version 6.3 (updated February 2022). Cochrane, 2022. Disponível em: www.training.cochrane.org/handbook.

3. Deeks JJ, Higgins JPT, Altman DG (editors). Chapter 10: Analysing data and undertaking meta-analyses. In: Higgins JPT, Thomas J, Chandler J, Cumpston M, Li T, Page MJ, Welch VA (editors). Cochrane Handbook for Systematic Reviews of Interventions version 6.3 (updated February 2022). Cochrane, 2022. Disponível em: www.training.cochrane.org/handbook.

4. Schünemann HJ, Higgins JPT, Vist GE, Glasziou P, Akl EA, Skoetz N, Guyatt GH. Chapter 14: Completing ‘Summary of findings’ tables and grading the certainty of the evidence. In: Higgins JPT, Thomas J, Chandler J, Cumpston M, Li T, Page MJ, Welch VA (editors). Cochrane Handbook for Systematic Reviews of Interventions version 6.3 (updated February 2022). Cochrane, 2022. Disponível em: www.training.cochrane.org/handbook.

**Annex 1** Characteristics of included studies and risk of bias table.

| **Sujata, 2019** | |
| --- | --- |
| **Method** | Study design: Randomized controlled trial  Study grouping: Parallel group (two arms)  Recruitment: October 2016 to May 2017  Setting: Hospital  Follow-up duration: 3 hours |
| **Participants** | Number of randomized participants: 50 participants  Number of participants analyzed: 49 participants; propofol group: 25 participants; sevoflurane group: 24 participants  Number of losses to follow-up: 1 participant in the sevoflurane group  Baseline characteristics  Propofol:  • Age: 62.88 ± 8.14 years  • Sex: Men - 24/25  • Weight: 72.56 ± 9.78 kg  Sevoflurane:  • Age: 65.33 ± 8.51 years  • Sex: Men - 23/24  • Weight: 78.54 ± 14.84 kg  Inclusion criteria: Patients undergoing any robotic-assisted laparoscopic pelvic surgery. |
| **Intervention** | Characteristics of interventions  Propofol (n = 25 – analyzed)  • Dose: 8 mg/kg/h and adjusted later to achieve a BIS score of 40 -60  • Administration: continuous intravenous infusion  Sevoflurane (n = 24 – analyzed)  • Dose: inspired concentration to maintain a BIS score of 40-60  • Administration: inhalation |
| **Outcomes** | Intracranial pressure (optic nerve sheath diameter)  • Outcome type: Continuous  • Reporting: Complete  • Range: up to 5 mm  • Unit of measurement: millimeters  • Direction: smaller = better |
| **Identification** | Country: India  Primary Author: Nambiath Sujata  Institution: Max Super Speciality Hospital  Email: drnambiath@yahoo.com  Address: Department of Anesthesia and Pain Management, Max Hospital, No. 1 Press Enclave Road, Saket, New Delhi 110017, India |
| **Notes** | Sponsor: None  Conflicts of interest: All authors have declared no conflicts of interest  Comments: None |

| **Sujata, 2019 – Risk of bias** | | | |
| --- | --- | --- | --- |
| **Domain** | **Signaling issue** | **Response** | **Comments** |
| **Bias due to the randomization process** | 1.1 Was the allocation sequence random? | PY | **Citation:** *"After study inclusion, the subjects were randomized by the sealed envelope method into two groups".*  **Comments:** Probably done. |
|  | 1.2 Was the allocation sequence concealed until participants were enrolled and assigned to interventions? | Y |  |
|  | 1.3 Do baseline differences between intervention groups suggest a problem with the randomization process? | N | **Citation:** *"The patient demographics in the two groups were similar with no statistical difference in age or weight. The baseline mean arterial blood pressure and baseline ONSD were also comparable between the two groups (Table 2)."*  **Comments:** There was no difference in the baseline. |
|  | **Judgment of risk of bias** | **Low** |  |
| **Bias due to deviation from planned interventions** | 2.1. Were participants aware of their assigned intervention during the study? | N | **Citação:** *"An infusion pump containing propofol or normal saline as per group allocation was connected to all subjects. The pump and the vaporiser was covered with a sterile cloth for purposes of blinding."*  **Comentário:** Probably done. |
|  | 2.2. Were caregivers and people delivering the interventions aware of the intervention assigned to participants during the study? | N |  |
|  | 2.3. If Y/PY/NI to 2.1 or 2.2: Were there deviations from the intended intervention that arose because of the experimental context? | NE |  |
|  | 2.4 If Y/PY to 2.3: Were these deviations likely to affect the outcome? | NE |  |
|  | 2.5. If Y/PY/Ni to 2.4: Were these deviations from the intended intervention balanced across groups? | NE |  |
|  | 2.6 Was an appropriate analysis used to estimate the effect of assignment to the intervention? | N | Comment: Analysis performed for participants who completed the planned follow-ups. |
|  | 2.7 If N/PN/NI for 2.6: Was there potential for a substantial impact (on outcome) from failure to analyze participants in the group to which they were randomized? | N | Comment: The loss was balanced between the groups. |
|  | **Judgment of risk of bias** | **Some concerns** |  |
| **Bias due to loss of outcome data** | 3.1 Were data for this outcome available for all, or nearly all, randomized participants? | Y | Comment: Data available for 98% of randomized participants. |
|  | 3.2 If N/PN/NI for 3.1: Is there evidence that the result was not biased by missing outcome data? | NE |  |
|  | 3.3 If N/PN for 3.2: Could the lack of outcome depend on its true value? | NE |  |
|  | 3.4 If Y/PY/NI for 3.3: Is it likely that the missing outcome depended on its true value? | NE |  |
|  | **Judgment of risk of bias** | **Low** |  |
| **Bias in outcome measurement** | 4.1 Was the outcome measurement method inappropriate? | N | Comment: Appropriate methods were used. |
|  | 4.2 Did the measurement or determination of the outcome differ between the intervention groups? | N | Comment: There was no difference between the groups in the way of analyzing. |
|  | 4.3 Were outcome assessors aware of the intervention received by study participants? | N | **Citation:** *"The ONSD of both eyes were measured separately at fixed time points (Table 1) during the surgery using ultrasound by two independent anesthesiologists who were experienced in ocular scans and blinded to the group allocation. These observers were not involved in patient recruitment, randomisation, group allocation or data analysis."*  **Comments:** Probably done. |
|  | 4.4 If S/PS/SI to 4.3: Could the outcome assessment have been influenced by knowledge of the intervention received? | NE |  |
|  | 4.5 If S/PS/SI to 4.4: Is it likely that the outcome assessment was influenced by knowledge of the intervention received? | NE |  |
|  | **Judgment of risk of bias** | **Low** |  |
| **Bias in selection of reported outcome** | 5.1 Were the data that produced this outcome analyzed according to a prespecified analysis plan that was finalized before unmasked outcome data were available for analysis? | N | **Citation:** *"The trial was registered prior to patient enrollment at http://ctri.nic.in (REF/2016/11/012713, Principal investigator: Nambiath Sujata, Date of registration: 12th October 2017)."*  **Comments:** The record found for the present study was made retrospectively in a different issue than the one published (CTRI/2017/10/010068). |
|  | 5.2 ... multiple eligible outcome measures (e.g., scales, definitions, time points) within the outcome domain? | N | Comment: There was no selection of reported scales. |
|  | 5.3 ... multiple eligible analyses of the data? | N | Comment: There was no selection of specific analyses reported. |
|  | **Judgment of risk of bias** | **Some concerns** |  |
| **General Bias Risk** | **Judgment of risk of bias** | **Some concerns** |  |

Y, Yes; PY, Probably Yes; N, Not; PN, Probably Not; NI, No Information; NE, Not Evaluate.

| **Geng, 2021** | |
| --- | --- |
| **Method** | Study design: Randomized controlled trial  Study grouping: Parallel group (two arms)  Recruitment: February 2018 to June 2020  Setting: Hospital  Follow-up duration: 2 hours |
| **Participants** | Number of participants randomized: 116 participants  Number of participants analyzed: 110 participants; propofol group: 55 participants; sevoflurane group: 55 participants  Number of losses to follow-up: 3 participants in the propofol group and 3 participants in the sevoflurane group  Baseline characteristics  Propofol:  • Age: 40.53 ± 11.08 years  • Weight: 59 (54.5‒63) kg  • Height: 161.18 ± 4.2 cm  • BMI: 22.74 ± 2.28 kg/m^2^  Sevoflurane:  • Age: 41.15 ± 10.26 years  • Weight: 56 (51.9‒60) kg  • Height: 159.79 ± 4.5 cm  • BMI: 22.31 ± 2.15 kg/m^2^  Inclusion criteria: Women classified as class I‒II according to the standards and guidelines of the American Society of Anesthesiologists (ASA) and undergoing elective laparoscopic gynecologic surgery under general anesthesia for an estimated surgical time > 2h. |
| **Intervention** | Characteristics of interventions  Propofol (n = 55 – analyzed)  • Dose: 4 µg/mL for induction and 3.2 µg/mL for maintenance to achieve a BIS score of 40‒60  • Administration: continuous intravenous infusion  Sevoflurane (n = 55 – analyzed)  • Dose: minimum alveolar concentration of 1‒1.5 in 50% oxygen/air to maintain a BIS score of 40‒60  Administration: inhalation |
| **Outcomes** | Intracranial pressure (optic nerve sheath diameter)  • Outcome type: Continuous  • Reporting: Complete  • Range: up to 5 mm  • Unit of measurement: millimeters  • Direction: smaller = better |
| **Identification** | Country: China  Main author: Weilian Geng  Institution: Obstetrics and Gynecology Hospital of Fudan University  E-mail: drhuangsq@163.com  Address: Department of Anesthesia, Obstetrics and Gynecology Hospital of Fudan University, nº 128, Shenyang RD, Yangpu district, Shanghai 200090, China |
| **Notes** | Sponsor: None  Conflicts of interest: All authors have declared no conflicts of interest  Comments: None |

| **Geng, 2021 – Risk of bias** | | | |
| --- | --- | --- | --- |
| **Domain** | **Signaling issue** | **Response** | **Comments** |
| **Bias due to the randomization process** | 1.1 Was the allocation sequence random? | Y | **Citation:** *"The patients were randomly divided into the propofol group (Group P) or the sevoflurane group (Group S). Patients were randomized in a 1:1 ratio occurred by computerized sequence generation. An anesthesiologist, who was not involved in the study, created sealed opaque envelopes in which groupings were written randomized. Envelopes were opened in sequential order only after a patient had signed the consent form".*  **Comment:** Probably done |
|  | 1.2 Was the allocation sequence concealed until participants were enrolled and assigned to interventions? | Y |  |
|  | 1.3 Do baseline differences between intervention groups suggest a problem with the randomization process? | N | **Comment:** There was no difference in the baseline. |
|  | **Judgment of risk of bias** | **Low** |  |
| **Bias due to deviation from planned interventions** | 2.1. Were participants aware of their assigned intervention during the study? | NI | Comment: No information on masking of participants and caregivers |
|  | 2.2. Were caregivers and people delivering the interventions aware of the intervention assigned to participants during the study? | NI |  |
|  | 2.3. If Y/PY/NI to 2.1 or 2.2: Were there deviations from the intended intervention that arose because of the experimental context? | N | Comment: There were no deviations from the planned interventions. |
|  | 2.4 If Y/PY to 2.3: Were these deviations likely to affect the outcome? | NE |  |
|  | 2.5. If Y/PY/Ni to 2.4: Were these deviations from the intended intervention balanced across groups? | NE |  |
|  | 2.6 Was an appropriate analysis used to estimate the effect of assignment to the intervention? | N | Comment: Analysis performed for participants who completed the planned follow-ups. |
|  | 2.7 If N/PN/NI for 2.6: Was there potential for a substantial impact (on outcome) from failure to analyze participants in the group to which they were randomized? | N | Comment: The loss was balanced between the groups. |
|  | **Judgment of risk of bias** | **Some concerns** |  |
| **Bias due to loss of outcome data** | 3.1 Were data for this outcome available for all, or nearly all, randomized participants? | Y | Comment: Data available for 94.8% of randomized participants |
|  | 3.2 If N/PN/NI for 3.1: Is there evidence that the result was not biased by missing outcome data? | NE |  |
|  | 3.3 If N/PN for 3.2: Could the lack of outcome depend on its true value? | NE |  |
|  | 3.4 If Y/PY/NI for 3.3: Is it likely that the missing outcome depended on its true value? | NE |  |
|  | **Judgment of risk of bias** | **Low** |  |
| **Bias in outcome measurement** | 4.1 Was the outcome measurement method inappropriate? | N | Comment: Appropriate methods were used. |
|  | 4.2 Did the measurement or determination of the outcome differ between the intervention groups? | N | Comment: There was no difference between the groups in the way of analyzing. |
|  | 4.3 Were outcome assessors aware of the intervention received by study participants? | N | ***Citation:*** *"A trained anaesthesiologist who was blinded for group allocation took the images of optic nerve sheath in all patients in this study, and ONSD was measured based on stored images by an experienced ultrasound doctor, and the average value was taken, with an accuracy of 0.1 mm".*  Comment: Probably done |
|  | 4.4 If S/PS/SI to 4.3: Could the outcome assessment have been influenced by knowledge of the intervention received? | NE |  |
|  | 4.5 If S/PS/SI to 4.4: Is it likely that the outcome assessment was influenced by knowledge of the intervention received? | NE |  |
|  | **Judgment of risk of bias** | **Low** |  |
| **Bias in selection of reported outcome** | 5.1 Were the data that produced this outcome analyzed according to a prespecified analysis plan that was finalized before unmasked outcome data were available for analysis? | Y | **Citation: “**The study was registered with clinicaltrials.gov (NCT03498235)."  Comment: The outcome described was pre-planned |
|  | 5.2 ... multiple eligible outcome measures (e.g., scales, definitions, time points) within the outcome domain? | N | Comment: There was no selection of reported scales. |
|  | 5.3 ... multiple eligible analyses of the data? | N | Comment: There was no selection of specific analyses reported. |
|  | **Judgment of risk of bias** | **Low** |  |
| **General Bias Risk** | **Judgment of risk of bias** | **Some concerns** |  |

Y, Yes; PY, Probably Yes; N, Not; PN, Probably Not; NI, No Information; NE, Not Evaluate.

| **Kim, 2019** | |
| --- | --- |
| **Method** | Study design: Randomized controlled clinical trial  Study group: Parallel group (two arms)  Recruitment: Not provided  Setting: Hospital  Follow-up duration: 1 hour and 30 minutes |
| **Participants** | Number of participants randomized: 32 participants  Number of participants analyzed: 32 participants; propofol group: 16 participants; sevoflurane group: 16 participants  Number of losses to follow-up: No losses  Baseline characteristics  Propofol:  • Age: 64.38 ± 7.86 years  • Weight: 69.38 ± 10.25 kg  • Height: 167.63 ± 5.94 cm  Sevoflurane:  • Age: 68.44 ± 7.97 years  • Weight: 66.69 ± 8.65 kg  • Height: 166.94 ± 4.42 cm  Inclusion criteria: Male patients between 19 and 79 years of age with an American Society of Anesthesiologists (ASA) physical status of I‒III who underwent robot-assisted laparoscopic radical prostatectomy. |
| **Intervention** | Characteristics of interventions  Propofol (n = 16 – analyzed)  • Dose: maintenance to achieve a BIS score of 40‒60 (mean dose used not reported)  • Administration: continuous intravenous infusion  Sevoflurane (n = 16 – analyzed)  • Dose: minimum alveolar concentration of 1‒2 vol/% to maintain a BIS score of 40‒60  Administration: inhalation |
| **Outcomes** | Intracranial pressure (optic nerve sheath diameter)  • Outcome type: Continuous  • Reporting: Complete  • Range: up to 5 mm  • Unit of measurement: millimeters  • Direction: smaller = better |
| **Identification** | **País:** República da Coreia  **Autor principal:** Yanghyun Kim  **Instituição:** Department of Anesthesia and Pain Medicine, National Cancer Center  **E-mail:** 11466@ncc.re.kr  **Endereço:** Não informado |
| **Notes** | Sponsor: None  Conflicts of interest: All authors have declared no conflicts of interest  Comments: None |

| **Kim, 2019 – Risk of bias** | | | |
| --- | --- | --- | --- |
| **Domain** | **Signaling issue** | **Response** | **Comments** |
| **Bias due to the randomization process** | 1.1 Was the allocation sequence random? | Y | **Citation:** *"The random assignments were generated by using a computer with block sizes of 2 and 4 with a 1:1 assignment using the random block size permutation method."*  **Citation:** *"The vaporizer and TCI pump were concealed from the anesthesiologists by using a screen."*  **Comment:** Problaby done. |
|  | 1.2 Was the allocation sequence concealed until participants were enrolled and assigned to interventions? | PY |  |
|  | 1.3 Do baseline differences between intervention groups suggest a problem with the randomization process? | N | **Citation:** *"No significant differences were observed in the demographic data of the patients, surgery time, or intraoperative variables, including hemodynamic and respiratory variables at any of the time points."*  **Comment:** There were no differences in the baseline. |
|  | **Judgment of risk of bias** | **Low** |  |
| **Bias due to deviation from planned interventions** | 2.1. Were participants aware of their assigned intervention during the study? | PN | **Citation:** *"The vaporizer and TCI pump were concealed from the anesthesiologists by using a screen."*  **Comment:** Probably done. |
|  | 2.2. Were caregivers and people delivering the interventions aware of the intervention assigned to participants during the study? | N |  |
|  | 2.3. If Y/PY/NI to 2.1 or 2.2: Were there deviations from the intended intervention that arose because of the experimental context? | NE |  |
|  | 2.4 If Y/PY to 2.3: Were these deviations likely to affect the outcome? | NE |  |
|  | 2.5. If Y/PY/Ni to 2.4: Were these deviations from the intended intervention balanced across groups? | NE |  |
|  | 2.6 Was an appropriate analysis used to estimate the effect of assignment to the intervention? | Y | Comment: All randomized participants were analyzed. |
|  | 2.7 If N/PN/NI for 2.6: Was there potential for a substantial impact (on outcome) from failure to analyze participants in the group to which they were randomized? | NE |  |
|  | **Judgment of risk of bias** | **Low** |  |
| **Bias due to loss of outcome data** | 3.1 Were data for this outcome available for all, or nearly all, randomized participants? | Y | Comment: All randomized participants were analyzed. |
|  | 3.2 If N/PN/NI for 3.1: Is there evidence that the result was not biased by missing outcome data? | NE |  |
|  | 3.3 If N/PN for 3.2: Could the lack of outcome depend on its true value? | NE |  |
|  | 3.4 If Y/PY/NI for 3.3: Is it likely that the missing outcome depended on its true value? | NE |  |
|  | **Judgment of risk of bias** | **Low** |  |
| **Bias in outcome measurement** | 4.1 Was the outcome measurement method inappropriate? | N | Comment: Appropriate methods were used. |
|  | 4.2 Did the measurement or determination of the outcome differ between the intervention groups? | N | Comment: There was no difference between the groups in the way of analyzing. |
|  | 4.3 Were outcome assessors aware of the intervention received by study participants? | N | **Citation:** *"The blinded anesthesiologists measured ONSD, evaluated as described previously."*  **Comment:** Probably done |
|  | 4.4 If S/PS/SI to 4.3: Could the outcome assessment have been influenced by knowledge of the intervention received? | NE |  |
|  | 4.5 If S/PS/SI to 4.4: Is it likely that the outcome assessment was influenced by knowledge of the intervention received? | NE |  |
|  | **Judgment of risk of bias** | **Low** |  |
| **Bias in selection of reported outcome** | 5.1 Were the data that produced this outcome analyzed according to a prespecified analysis plan that was finalized before unmasked outcome data were available for analysis? | Y | **Citation:** *"It is registered with the Korean Clinical Trials Registry (CRiS, http://cris.nih.go.kr, 9/11/2018, KCT 0003332)."*  Comment: The outcome described was pre-planned. |
|  | 5.2 ... multiple eligible outcome measures (e.g., scales, definitions, time points) within the outcome domain? | N | Comment: There was no selection of reported scales. |
|  | 5.3 ... multiple eligible analyses of the data? | N | Comment: There was no selection of specific analyses reported. |
|  | **Judgment of risk of bias** | **Low** |  |
| **General Bias Risk** | **Judgment of risk of bias** | **Low** |  |

Y, Yes; PY, Probably Yes; N, Not; PN, Probably Not; NI, No Information; NE, Not Evaluate.

| **Yu, 2018** | |
| --- | --- |
| **Method** | Study design: Randomized controlled clinical trial  Study group: Parallel group (two arms)  Recruitment: Not provided  Setting: Hospital  Follow-up duration: 1 hour |
| **Participants** | Number of randomized participants: 36 participants  Number of participants analyzed: 36 participants; propofol group: 18 participants; sevoflurane group: 18 participants  Number of losses to follow-up: No losses  Baseline characteristics  Propofol:  • Age: 66.1 ± 7.2 years  • Weight: 72.3 ± 6.5 kg  • Height: 166 ± 4 cm  • BMI: 26.1 ± 1.9 cm  Sevoflurane:  • Age: 63.6 ± 7.9 years  • Weight: 69.8 ± 10.6 kg  • Height: 165.6 ± 7.6 cm  • BMI: 25.4 ± 2.7 kg/m^2^  Inclusion criteria: Patients who were scheduled for a robotic-assisted laparoscopic prostatectomy using the da Vinci™ robotic system (Intuitive Surgical, Inc., Sunnyvale, CA, USA) |
| **Intervention** | Characteristics of interventions  Propofol (n = 18 – analyzed)  • Dose: 1.5‒3 µg/mL to achieve a BIS score of 40‒60  • Administration: continuous intravenous infusion  Sevoflurane (n = 18 – analyzed)  • Dose: 1‒2 vol/% to maintain a BIS score of 40‒60  Administration: inhalation |
| **Outcomes** | Intracranial pressure (optic nerve sheath diameter)  • Outcome type: Continuous  • Reporting: Complete  • Range: up to 5 mm  • Unit of measurement: millimeters  • Direction: smaller = better |
| **Identification** | Country: Republic of Korea  Primary author: Jihion Yu  Institution: Asan Medical Center  E-mail: sscho@hallym.or.kr; kyk@amc.seoul.kr  Address: Department of Anesthesiology and Pain Medicine, Asan Medical Center, University of Ulsan College of Medicine, 88, Olympic-ro 43-gil, Songpa-gu, Seoul 05505, Republic of Korea |
| **Notes** | Sponsor: Asan Medical Center  Conflicts of interest: All authors have declared no conflicts of interest  Comments: None |

| **Yu, 2018 – Risk of bias** | | | |
| --- | --- | --- | --- |
| **Domain** | **Signaling issue** | **Response** | **Comments** |
| **Bias due to the randomization process** | 1.1 Was the allocation sequence random? | Y | **Citation:** *"Patients were randomly assigned to two groups using web-based randomization software (Random Allocation Software version 1.0, Isfahan University of Medical Sciences, Isfahan, Iran). We used block randomization with random block sizes of 6 and an allocation ratio of 1:1. One investigator kept sealed envelopes labeled with sequential study numbers, which were opened just before induction of anesthesia. The investigator performed total intravenous anesthesia with propofol (propofol group) or inhalation anesthesia with sevoflurane (sevoflurane group) according to a randomized table."*  **Comment:** Probably done |
|  | 1.2 Was the allocation sequence concealed until participants were enrolled and assigned to interventions? | Y |  |
|  | 1.3 Do baseline differences between intervention groups suggest a problem with the randomization process? | N | **Citation:** *"Demographic and intraoperative data were not significantly different between the two groups (Table 1)."* |
|  | **Judgment of risk of bias** | **Low** | Comment: There was no difference at baseline. |
| **Bias due to deviation from planned interventions** | 2.1. Were participants aware of their assigned intervention during the study? | N | **Citation:** *"The ventilator screen and the syringes of medications are concealed. We also prepared concealed syringes of normal saline in the sevoflurane group, indistinguishable from the outside."*  **Comment:** Probably done |
|  | 2.2. Were caregivers and people delivering the interventions aware of the intervention assigned to participants during the study? | N |  |
|  | 2.3. If Y/PY/NI to 2.1 or 2.2: Were there deviations from the intended intervention that arose because of the experimental context? | NE |  |
|  | 2.4 If Y/PY to 2.3: Were these deviations likely to affect the outcome? | NE |  |
|  | 2.5. If Y/PY/Ni to 2.4: Were these deviations from the intended intervention balanced across groups? | NE |  |
|  | 2.6 Was an appropriate analysis used to estimate the effect of assignment to the intervention? | Y | Comment: All randomized participants were analyzed. |
|  | 2.7 If N/PN/NI for 2.6: Was there potential for a substantial impact (on outcome) from failure to analyze participants in the group to which they were randomized? | NE |  |
|  | **Judgment of risk of bias** | **Low** |  |
| **Bias due to loss of outcome data** | 3.1 Were data for this outcome available for all, or nearly all, randomized participants? | Y | Comment: All randomized participants were analyzed. |
|  | 3.2 If N/PN/NI for 3.1: Is there evidence that the result was not biased by missing outcome data? | NE |  |
|  | 3.3 If N/PN for 3.2: Could the lack of outcome depend on its true value? | NE |  |
|  | 3.4 If Y/PY/NI for 3.3: Is it likely that the missing outcome depended on its true value? | NE |  |
|  | **Judgment of risk of bias** | **Low** |  |
| **Bias in outcome measurement** | 4.1 Was the outcome measurement method inappropriate? | N | Comment: Appropriate methods were used |
|  | 4.2 Did the measurement or determination of the outcome differ between the intervention groups? | N | Comment: There was no difference between the groups in the way of analyzing. |
|  | 4.3 Were outcome assessors aware of the intervention received by study participants? | N | **Citation:** *"The investigators who analyzed the data did not know the group. The investigators were blinded to the allocation of treatment until data analysis was complete."*  **Comment:** Probably done. |
|  | 4.4 If S/PS/SI to 4.3: Could the outcome assessment have been influenced by knowledge of the intervention received? | NE |  |
|  | 4.5 If S/PS/SI to 4.4: Is it likely that the outcome assessment was influenced by knowledge of the intervention received? | NE |  |
|  | **Judgment of risk of bias** | **Low** |  |
| **Bias in selection of reported outcome** | 5.1 Were the data that produced this outcome analyzed according to a prespecified analysis plan that was finalized before unmasked outcome data were available for analysis? | Y | **Citation:** *"This study was registered with ClinicalTrials.gov (NCT03271502)."*  Comment: The outcome described was pre-planned |
|  | 5.2 ... multiple eligible outcome measures (e.g., scales, definitions, time points) within the outcome domain? | N | Comment: There was no selection of reported scales |
|  | 5.3 ... multiple eligible analyses of the data? | N | Comment: There was no selection of specific analyses reported. |
|  | **Judgment of risk of bias** | **Low** |  |
| **General Bias Risk** | **Judgment of risk of bias** | **Low** |  |

Y – Yes; PY – Probably Yes; N – Not; PN – Probably Not; NI – No Information; NE - not evaluate

***Lee, 2019***

| **Method** | Study design: Randomized controlled clinical trial  Study group: Parallel group (two arms)  Recruitment: July 2018 to October 2018  Setting: Hospital  Follow-up duration: 30 minutes |
| --- | --- |
| **Participants** | Number of participants randomized: 36 participants  Number of participants analyzed: 36 participants; propofol group: 18 participants; sevoflurane group: 18 participants  Number of losses to follow-up: No losses  Baseline characteristics  Propofol:  • Age: 45 ± 13.8 years  • Weight: 58 ± 6.8 kg  • Height: 161 ± 5.7 cm  Sevoflurane:  • Age: 44 ± 11.9 years  • Weight: 56 ± 9.8 kg  • Height: 158 ± 5.6 cm  Inclusion criteria: Female patients with American Society of Anesthesiologists physical status I-II, aged 20 to 65 years, who underwent elective robotic or laparoscopic gynecologic surgery, such as robotic-assisted myomectomy, robotic or laparoscopic total hysterectomy, robotic-assisted sacrocolpopexy, and ovarian cystectomy |
| **Intervention** | Characteristics of interventions  Propofol (n = 36 – analyzed)  • Dose: 2 mg/kg (3 µg/ml) to achieve a BIS score of 40-60  • Administration: continuous intravenous infusion  Sevoflurane (n = 36 – analyzed)  • Dose: 2 vol/% to maintain a BIS score of 40-60  Administration: inhalation |
| **Outcomes** | Intracranial pressure (optic nerve sheath diameter)  • Outcome type: Continuous  • Reporting: Complete  • Range: up to 5 mm  • Unit of measurement: millimeters  • Direction: smaller = better |
| **Identification** | **Country:** Corean Republic  **Principal author:** Youn Young Lee  **Institution:** Department of Anesthesiology and Pain Medicine, Ewha Womans University College of Medicine  **E-mail:** [leehee@ewha.ac.k](mailto:leehee@ewha.ac.k) |
| **Notes** | Sponsor: Not disclosed  Conflicts of interest: No potential conflicts of interest relevant to this article were reported.  Comments: None. |

***Lee, 2019 – Risk of bias***

| **Domain** | **Signaling issue** | **Response** | **Comments** |
| --- | --- | --- | --- |
| **Bias due to the randomization process** | 1.1 Was the allocation sequence random? | Y | **Citation:** *"After study inclusion, the subjects were randomized by the sealed envelope method into two groups".*  **Comments:** Probably done. |
|  | 1.2 Was the allocation sequence concealed until participants were enrolled and assigned to interventions? | NI |  |
|  | 1.3 Do baseline differences between intervention groups suggest a problem with the randomization process? | N | **Citation:** *"The patient demographics in the two groups were similar with no statistical difference in age or weight. The baseline mean arterial blood pressure and baseline ONSD were also comparable between the two groups (Table 2)."*  **Comments:** There was no difference in the baseline. |
|  | **Judgment of risk of bias** | **Some concerns** |  |
| **Bias due to deviation from planned interventions** | 2.1. Were participants aware of their assigned intervention during the study? | NI | Comment: There was no description of participant masking and due to the nature of the interventions it is likely that researchers were not masked during the use of the interventions. |
|  | 2.2. Were caregivers and people delivering the interventions aware of the intervention assigned to participants during the study? | PY |  |
|  | 2.3. If Y/PY/NI to 2.1 or 2.2: Were there deviations from the intended intervention that arose because of the experimental context? | PN | Comment: There was no deviation from the planned interventions |
|  | 2.4 If Y/PY to 2.3: Were these deviations likely to affect the outcome? | NE |  |
|  | 2.5. If Y/PY/Ni to 2.4: Were these deviations from the intended intervention balanced across groups? | NE |  |
|  | 2.6 Was an appropriate analysis used to estimate the effect of assignment to the intervention? | N | Comment: Analysis performed for participants who completed the planned follow-ups. |
|  | 2.7 If N/PN/NI for 2.6: Was there potential for a substantial impact (on outcome) from failure to analyze participants in the group to which they were randomized? | N | Comment: The loss was balanced between the groups. |
|  | **Judgment of risk of bias** | **Some concerns** |  |
| **Bias due to loss of outcome data** | 3.1 Were data for this outcome available for all, or nearly all, randomized participants? | Y | Comment: Data available for 98% of randomized participants. |
|  | 3.2 If N/PN/NI for 3.1: Is there evidence that the result was not biased by missing outcome data? | NE |  |
|  | 3.3 If N/PN for 3.2: Could the lack of outcome depend on its true value? | NE |  |
|  | 3.4 If Y/PY/NI for 3.3: Is it likely that the missing outcome depended on its true value? | NE |  |
|  | **Judgment of risk of bias** | **Low** |  |
| **Bias in outcome measurement** | 4.1 Was the outcome measurement method inappropriate? | N | Comment: Appropriate methods were used |
|  | 4.2 Did the measurement or determination of the outcome differ between the intervention groups? | N | Comment: There was no difference between the groups in the way of analyzing |
|  | 4.3 Were outcome assessors aware of the intervention received by study participants? | NI | Comment: There is no information on reviewer masking. |
|  | 4.4 If S/PS/SI to 4.3: Could the outcome assessment have been influenced by knowledge of the intervention received? | PY | Comment: Although there is a possibility of influence by the evaluators in the case of knowledge of the interventions, the results do not appear to have been influenced. |
|  | 4.5 If S/PS/SI to 4.4: Is it likely that the outcome assessment was influenced by knowledge of the intervention received? | PN |  |
|  | **Judgment of risk of bias** | **Some concerns** |  |
| **Bias in selection of reported outcome** | 5.1 Were the data that produced this outcome analyzed according to a prespecified analysis plan that was finalized before unmasked outcome data were available for analysis? | NI | Comment: There is no information about protocol registration. |
|  | 5.2 Multiple eligible outcome measures (e.g., scales, definitions, time points) within the outcome domain? | N | Comment: There was no selection of reported scales |
|  | 5.3 Multiple eligible analyses of the data? | N | Comment: There was no selection of specific analyses reported. |
|  | **Judgment of risk of bias** | **Some concerns** |  |
| **General Bias Risk** | **Judgment of risk of bias** | **Some concerns** |  |

**Legend:** Y – Yes; PY – Probably Yes; N – Not; PN – Probably Not; NI – No Information; NE - not evaluate
